# Non-pharmacological interventions to manage psychological distress in patients living with cancer: a systematic review

**DOI:** 10.1186/s12904-023-01202-8

**Published:** 2023-07-06

**Authors:** Carole A. Paley, Jason W. Boland, Martina Santarelli, Fliss E. M. Murtagh, Lucy Ziegler, Emma J. Chapman

**Affiliations:** 1 University of Leeds, Academic Unit of Palliative Care, Leeds, UK; 2 Wolfson Palliative Care Research Centre, Hull York Medical School, University of Hull, Hull, UK

**Keywords:** Distress; psychological distress; psychological intervention; neoplasms; palliative care; psychosocial oncology

## Abstract

**Background:**

Psychological distress is common in patients with cancer; interfering with physical and psychological wellbeing, and hindering management of physical symptoms. Our aim was to systematically review published evidence on non-pharmacological interventions for cancer-related psychological distress, at all stages of the disease.

**Methods:**

We followed the Preferred Reporting Items for Systematic Reviews and Meta-Analyses (PRISMA) guidelines. The review was registered on PROSPERO (CRD42022311729). Searches were made using eight online databases to identify studies meeting our inclusion criteria.

Data were collected on outcome measures, modes of delivery, resources and evidence of efficacy. A meta-analysis was planned if data allowed. Quality was assessed using the Mixed Methods Appraisal Tool (MMAT).

**Results:**

Fifty-nine studies with 17,628 participants were included. One third of studies included mindfulness, talking or group therapies. Half of all studies reported statistically significant improvements in distress. Statistically significant intervention effects on distress were most prevalent for mindfulness techniques. Four of these mindfulness studies had moderate effect sizes (*d* = -0.71[95% CI: -1.04, -0.37] *p* < *0.001*) (*d* = -0.60 [95% CI: -3.44, -0.89] *p* < *0.001*) (*d* = -0.77 [CI: -0.146, -1.954] *p* < *0.01*) (*d* = -0.69 [CI: -0.18, -1.19] *p* = *0.008*) and one had a large effect size (*d* = -1.03 [95% CI: -1.51, -0.54] *p* < *0.001*). Heterogeneity of studies precluded meta-analysis. Study quality was variable and some had a high risk of bias.

**Conclusions:**

The majority of studies using a mindfulness intervention in this review are efficacious at alleviating distress. Mindfulness—including brief, self-administered interventions—merits further investigation, using adequately powered, high-quality studies.

**Systematic review registration:**

This systematic review is registered on PROSPERO, number CRD42022311729.

**Supplementary Information:**

The online version contains supplementary material available at 10.1186/s12904-023-01202-8.

## Background

Psychological distress is highly prevalent in patients living with cancer. Despite its identification and management being supported by a growing body of literature, there is still no universally shared understanding of the concept of distress [1]. The National Comprehensive Cancer Network (NCCN), in their recently revised guidance, define distress in cancer as ‘….a multifactorial unpleasant experience of a psychological (i.e., cognitive, behavioral, emotional), social, spiritual, and/or physical nature that may interfere with one’s ability to cope effectively with cancer, its physical symptoms, and its treatment’ [2]. The American Medical Association (AMA) characterises psychological distress as an inability to cope with the disease or its treatment, a lack of control and a condition distinct from anxiety and depression [3]. Distress has been proposed as the 6^th^ vital sign in cancer care by the International Psycho-Oncology Society [4, 5] and the AMA recommend that screening for, and treating, distress should become an integral part of care plans. They propose that ‘…. novel interventions to address distress must be developed and rigorously tested…’ [3].

Huda, et al. [1] and Ridner [6] attempted to produce conceptual models for psychological distress in advanced cancer using an adaptation of the Walker and Avant model [7]. They identified defining attributes of cancer-related distress, such as anxiety, depression, loss of hope and having to come to terms with a potential life-limiting disease. The resulting consequences of these attributes are on a continuum from positive to negative, but are frequently negative. These range from mild and infrequent mood disturbances, through to situations where friends and family become affected, symptoms are exacerbated and the patient experiences a loss of coping strategies [1, 6]. Cancer-related psychological distress may be complex and can be a barrier to effective management of symptoms such as fatigue, pain and breathlessness [8]. It is also detrimental to health-related behaviours which can result in an exacerbation of mental health issues such as stress, anxiety and depression [9, 10]. Distress also affects relationships between cancer patients, family members and carers [11–13]. 

Approximately 40% of patients with cancer suffer symptoms related to distress, with higher rates reported (58%) amongst patients receiving palliative care [14]. Alternatively, 52% of patients with cancer are reported to have high levels of psychological distress when defined as ≥ 5 on the Distress Thermometer (DT), accompanied by fatigue, sadness and sleep problems [15]. Despite this high prevalence, 71% of patients with ‘significant distress’ decline help; most commonly because they consider their condition was not severe enough or because they prefer to manage it themselves [16]. Patients with cancer who are distressed frequently refuse treatment for it [17], even though alleviating distress might facilitate more effective symptom management [8]. This might be due, in part, to the stigma associated with having a mental illness which can lead to social disapproval or diminished self-esteem at a time when it is possibly most needed [18].

The importance of screening for distress is increasingly recognised as important in cancer care [19]. However, as identified by Deshields, et al. [10] there is a lack of detail or consistency in currently available guidance. A systematic review published in 2018 by McCarter, et al., revealed a lack of robust evidence for effective strategies to improve the routine implementation of distress screening and referral for patients with cancer [20]. The review also identified a lack of training in distress screening amongst clinical staff. Importantly, it has been identified that distress changes significantly at key stages during the cancer trajectory [21], and suggested that screening measures at each key stage of the disease should be ongoing for patients at the time of diagnosis, during initial treatment, following treatment and at the time of recurrence [22].

More recently a new clinical pathway has been developed and tested for the screening, assessment and management of anxiety and depression in adult cancer patients (ADAPT CP), and this might also provide a useful tool for identifying psychological distress at key disease stages [23, 24].

It has been suggested that patients with cancer might benefit physically, as well as psychologically, from appropriate interventions for distress. Improvements in psychological and physical symptoms and in overall well-being were achieved in patients who were routinely screened for distress and received appropriate interventions [25]. Distress and physical symptoms, particularly fatigue and pain, have been shown to be interrelated in patients with malignant myelodysplastic syndromes [26].

A great deal of literature on the alleviation of distress, anxiety and depression in cancer has focused on the use of cognitive behavioural therapy (CBT) or combinations of therapies including CBT techniques, such as mindfulness-based cognitive therapy (MBCT) or acceptance and commitment therapy (ACT) [27]. However, systematic reviews often reveal small effect sizes and methodological shortcomings [28] and a review of reviews of psychological interventions for distress stated that there was a lack of systematically reviewed evidence of good quality [29].

A systematic review by Warth, et al., investigated the use of brief psychological interventions (four sessions or less, over fewer than 21 days) for improving psychological well-being in palliative care. Patients reported that these were effective in improving quality of life and in reducing emotional distress and existential suffering [30]. The most commonly reported techniques in this review were life review techniques and music therapy. Although the study was in patients nearing end of life, it is likely that such interventions will be relevant for cancer patients at earlier stages of the disease too. Another systematic review by Xunlin, et al. [31] looked at mindfulness-based stress reduction techniques for a variety of psychological symptoms and quality of life in breast cancer patients and found promising improvements in distress. Other reviews have focussed on mindfulness interventions alone and found some evidence of efficacy but clinical evidence was lacking [32, 33].

The available evidence suggests that there are many potential benefits in providing effective screening for cancer-related distress and implementing interventions to alleviate it. However, the systematic reviews and meta-analyses conducted to date have not considered distress in all types of cancer and at all stages of the disease and their inclusion criteria has been relatively narrow. Therefore, the research question, which provided the basis for our methodology, was to investigate what interventions were specifically used to manage cancer-related distress at all stages of active disease. The primary aim of our systematic review was to identify and synthesise randomised controlled trials (RCTs) and non-randomised controlled clinical trials (CCTs) investigating interventions specifically targeting cancer-related psychological distress in patients with any type or stage of the disease.

## Methods

This systematic review was registered on PROSPERO, number CRD42022311729.

### Criteria for considering studies for this review

For the purposes of this review, the definition of psychological distress is taken from the NCCN Guidelines [2].

#### Inclusion criteria

Using the PICOS framework, the following criteria were used:

- Population
  i.  adults (age ≥ 18 years) of whom > 50% have any type/stage of cancer, currently with active disease, in any setting
- Interventions
  i. non-pharmacological interventions aimed at alleviating psychological distress
- Comparators
  i. no treatment, usual care, treatment-as-usual, waiting list or active comparators
- Outcome Measures
  i. psychological distress as a primary outcome
- Study design
  i. RCTs and CCTs
  ii. Studies with primarily quantitative data, or studies with mixed-methodologies.

#### Exclusion criteria

##### Types of studies

i. Qualitative studies with no quantitative data
ii. Case studies, surveys, audits, and uncontrolled studies
iii. Protocols
iv. Systematic reviews or narrative reviews
v. Grey literature
vi. Letters, editorials, and conference abstracts.

##### Study populations

i. Animal studies
ii. Studies including > 50% of persons under the age of 18 years.
iii. Populations stated to be ‘cancer survivors’ or having undergone curative treatment (i.e., has either had cancer and is deemed to be cured, or has completed treatment and has no evidence of active disease).

We also excluded any studies not written in, or translated into English.

### Data sources

The following electronic databases were searched for articles published from 2002 to the present (2022):

- MEDLINE (via OVID)
- Web of Science
- Scopus
- CINAHL (via EBSCO)
- PubMed
- APA PsycINFO (OVID)
- AMED (OVID)
- CENTRAL (Cochrane)

Additional references were included from an initial scoping review if not identified during the main searches.

### Search strategy

All online search strategies are included in Appendix A (Supplementary information).

Reference lists of other systematic reviews were also screened against inclusion criteria.

The results of searches and screening were reported according to the Preferred Reporting Items for Systematic reviews and Meta-Analyses (PRISMA) guidelines [34, 35] (PRISMA checklist: Appendix B (Supplementary information)).

### Data management and synthesis

Management of data was achieved using the Covidence systematic review software [36]. Two independent reviewers (CP and EC) screened studies which met the eligibility criteria by title and abstract. Full-text review was carried out if studies were deemed eligible or where eligibility was unclear. Where reviewers disagreed on inclusion/exclusion, a third author acted as arbiter. Data collection was completed using a template created which was specifically designed for this review (Appendix C (Supplementary information)).

A narrative synthesis was planned. Clinically and statistically significant differences in distress due to the intervention would be reported for included studies. Where effect sizes and confidence intervals were not included in the study reports, these were calculated provided the necessary data were available. If data allowed, meta-analysis would be utilised to examine change in distress outcomes (effect size (Standard Mean Difference)) for different interventions. Further subgroup analysis was not planned.

### Quality and risk of bias assessment

Quality was assessed using the Mixed Methods Appraisal Tool (MMAT) [37] (Appendix D(a) (Supplementary information)). Three additional questions were added to enable further appraisal of overall methodological quality and risk of bias. These were: ‘Was attrition/exclusion data reported?’, ‘Were adverse events reported?’ and ‘Was an appropriate sample size calculation carried out?’ Reporting of attrition and adverse event are elements of risk of bias from selective reporting, as outlined by Higgins, et al. in the Cochrane Handbook [38]. The issue of sample size is the subject of much debate and for the purposes of meta-analysis it has been stated that individual studies should have arms of ≥ 200 participants, or pooled events of ≥ 500 otherwise they are at high risk of bias and likely to produce imprecise effect estimates [39, 40].

## Results

**Fig. 1:**
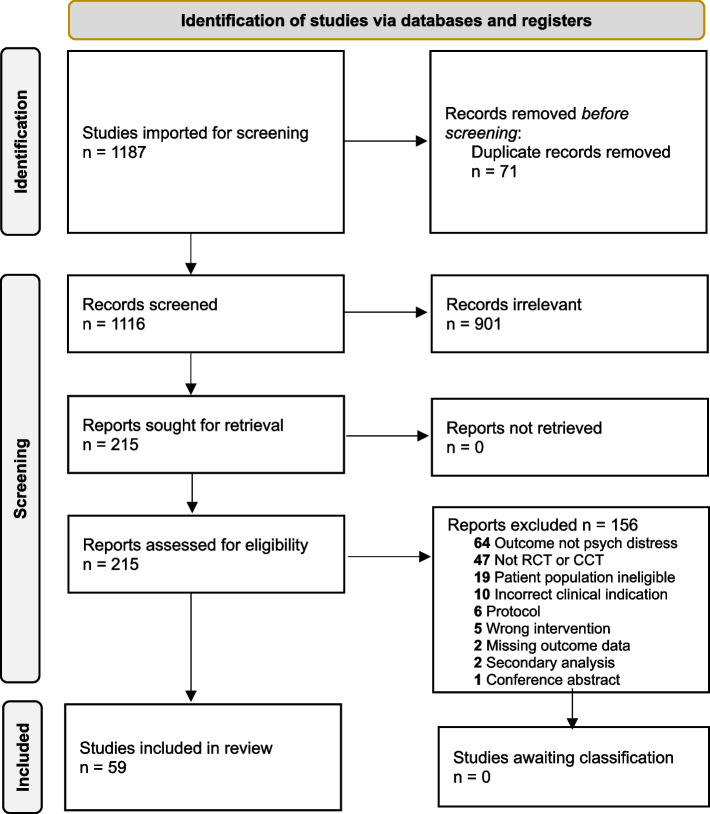
PRISMA flow diagram [34]

One thousand one hundred sixteen records were screened and fifty-nine studies with 17,628 participants were included. The literature screening process was recorded and illustrated according to PRISMA guidelines in the flow diagram below (Fig. 1).

### Characteristics of included studies

Table 1 summarises study characteristics, interventions and comparators, measures of distress used and whether the results were statistically significant. Effect sizes are included where these were given or could be calculated from the available data.

**Table 1:** General characteristics of included studies

General study information					Cancer type/spread		Study intervention characteristics				Evidence of efficacy
**Lead author name**	**Year of publication**	**Title**	**Study Design**	**No of particip-ants**	**Diagnosis (primary)**	**Metastatic spread (Y/N)**	**Intervention 1 Characteristic**	**Intervention 2 Characteristic**	**Comparator(s) Characteristic**	**Measure of distress**	**Statistical Significance** **(CIs all 95%)**
**Mindfulness interventions**											
Chambers, SK [41]	2017	Mindfulness-Based Cognitive Therapy in Advanced Prostate Cancer: A Randomized Controlled Trial	RCT	190 consented 189 randomised	Prostate Cancer (advanced)	Y	MBCT	N/A	Minimally enhanced usual care (Control)	BSI-18 and IES	Not sig
Chui, PL [42]	2021	Mindful Breathing Effects of a five-minute practice on perceived stress and mindfulness among patients with cancer	RCT	83	Any stage I-III cancer	N	Mindful Breathing	N/A	Standard Care	PSS-10	Not sig
Compen, F [43]	2018	Face-to-Face and Internet-Based Mindfulness-Based Cognitive Therapy Compared with Treatment as Usual in Reducing Psychological Distress in Patients with Cancer: A Multicenter Randomized Controlled Trial	RCT	245	Cancer of any type or stage	Y	MBCT (mindfulness-based cognitive therapy)	eMBCT	Treatment as Usual (TAU)	HADS	Sig reduction in distress post both interventions p < 0.001 Effect sizes MBCT: d = -0.45 (small) 95% CI: -0.83, -0.14 eMBCT: d = -0.71 (mod) 95% CI: -1.04, -0.37 [RCI also shows clinical effectiveness for both]
Liu, Z [44]	2022	A randomized clinical trial of guided self-help intervention based on mindfulness for patients with hepatocellular carcinoma: effects and mechanisms	RCT	122	Hepatocellular carcinoma	Not reported	Guided self-help mindfulness-based intervention (GSH-MBI)	N/A	Waiting list control (CG)	HADS	Sig reduction in distress post intervention *p* < 0.001 Effect sizes: T2: d = -0.49 *P* < 0.01 (small); CI: -3.13, -0.51 T3: d = -0.60 *P* < 0001 (mod); 95% CI: -3.44, -0.89
Milbury, K [45]	2020	A Mindfulness-Based Intervention as a Supportive Care Strategy for Patients with Metastatic Non-Small Cell Lung Cancer and their Spouses: Results of a Three-Arm Pilot Randomized Controlled Trial	RCT	150 (75 patients and 75 spouses)	Metastatic non-small cell lung cancer	Y	Couple-based meditation (CBM)	Supportive-expressive intervention (SE)	Usual Care (UC)	IES	Not sig
Ng, CG [46]	2016	The Effect of 5 Minutes of Mindful Breathing to the Perception of Distress and Physiological Responses in Palliative Care Cancer Patients: A Randomized Controlled Study	RCT	60	Any site	Y	Mindful Breathing (MB)	N/A	Normal Listening (Control)	DT	Sig reduction in distress post-intervention and 10 min after (T3) *p* < 0.01 Effect size: T3: d = -0.77 (mod)^a^ CI: -0.146, -1.954^b^
Park, S [47]	2020	Mindfulness-Based Cognitive Therapy for Psychological Distress, Fear of Cancer Recurrence, Fatigue, Spiritual Well-Being, and Quality of Life in Patients with Breast Cancer a Randomized Controlled Trial	RCT	74	Stage I-III breast cancer	N	Mindfulness-based cognitive therapy (MBCT)	N/A	Wait-list control	HADS (TMD)	Sig reduction in distress post-intervention *p* < 0.001 Effect sizes: T1: d = -1.17 (large) CI: -1.67, -0.68^b^ T2: d = -1.03 (large) CI: -1.51, -0.54^b^
Schellekens, MPJ [48]	2017	Mindfulness-based stress reduction added to care as usual for lung cancer patients and/or their partners: A multicentre randomized controlled trial	RCT	63 patients, 44 partners	Lung: non-small cell Small cell Mesothelioma -	Y	Mindfulness-based stress reduction (MBSR) + Care as Usual (CAU)	N/A	CAU (control)	HADS	Sig reduction in distress *p* = 0.008 post intervention Effect size: CAU + MBSR: d = -0.69 (mod); CI:- 0.18, -1.19
Wurtzen, H [49]	2015	Effect of mindfulness-based stress reduction on somatic symptoms, distress, mindfulness and spiritual wellbeing in women with breast cancer: Results of a randomized controlled trial	RCT	336	Breast cancer stage I-III	N (Stage I-III)	Mindfulness-based stress reduction (MBSR)	N/A	Usual care	SCL-90r	Sig reduction in distress post-intervention (*p* = 0.01), 6 months *p* < 0.001 and 12 months (*p* = 0.04) Effect size: *d* = -0.43 (small) CI not calculated as no SDs provided
**Talking/communication/CBT/group therapies**											
Acevedo-Ibarra, JN [50]	2019	Cognitive Behavioral Stress Management intervention in Mexican colorectal cancer patients: Pilot study	RCT	94 randomised	Colorectal cancer	N	CBSM	N/A	PE	HADS	Not sig
Andersen, BL [51]	2007	Distress reduction from a psychological intervention contributes to improved health for cancer patients	RCT	227	Breast cancer Stage II (90%) Stage III (10%)	Not reported	Coping strategies, muscle relaxation training, effective use of social support	N/A	Assessment only	POMS TMD	A sig 3-way interaction was found at 12-months depending upon initial levels of Cancer Stress *p* < 0.05. Sample and data same as Andersen (2004) below
Andersen, BL [52]	2004	Psychological, Behavioral, and Immune Changes After a Psychological Intervention: A Clinical Trial	RCT	227	Breast cancer stage II/III	N	Psychological intervention	N/A	Assessment only	POMS	POMS sig at *p* < 0.05. Sig 3-way interaction (*P* = .03). POMS TMD decr more in intervention arm than assessment arm (*p* = .04) for subjects with high initial cancer stress Full data not provided
Boesen, EH [53]	2011	Psychosocial group intervention for patients with primary breast cancer: A randomised trial	RCT	205	Breast cancer stage I-III	Y (in some pts)	Existential-cognitive group therapy	N/A	Not stated—assume treatment-as-usual	POMS	Not sig
Boesen, EH [54]	2005	Psychoeducational Intervention for Patients with Cutaneous Malignant Melanoma: A Replication Study	RCT	262	Cutaneous Malignant Melanoma	Y (various)	Group healthcare info and advice with relaxation and guided imagery in groups/at home	N/A	Control group	POMS	Not sig
Chambers, SK [55]	2018	Web-Delivered Cognitive Behavioral Therapy for Distressed Cancer Patients: Randomized Controlled Trial	RCT	163		Not recorded	CancerCope programme	N/A	Patient education website	BSI-18 and IES	Sig reduction in psych distress *p* = 0.04 and cancer- specific distress *p* = 0.02 at 8 weeks (not sig on ITT analysis) Effect size: d = 0.63 (mod): for cancer-specific distress CI: 0.15, 1.10
Hejazi, F [56]	2017	The Effect of a Communicational Program on Psychological Distress in the Elderly Suffering from Cancer	RCT	64	Any cancer	Not reported	Communicational program	N/A	Usual care	Kessler (K10)	Sig reduction in distress *p* < 0.001 post-intervention Effect size: d = –9.34^a^ (large) CI: -12.97, -5.71^b^
Manne, SL [57]	2017	A Comparison of Two Psychological Interventions for Newly-diagnosed Gynecological Cancer Patients	RCT	352	Gynaecological cancers	Y—31% of full sample	Communication-enhancing intervention (CCI)	Supportive counselling intervention (SC)	Usual care (UC)	IES	Not sig
Mertz BG [58]	2017	The effects of individually tailored nurse navigation for patients with newly diagnosed breast cancer: a randomized pilot study	RCT	50	Breast Cancer	Not reported	Screening and counselling (IG)	N/A	Standard care (CG)	DT	Sig reduction in distress at 12 months post-intervention *p* < 0.01 Effect sizes: 6 mth d = -0.38 CI: -1.95, 1.19 (not sig) 12 mth: d = -2.40 (large)^a^ CI: -4.02, -0.78^b^
Taylor, K [59]	2003	Psychological Adjustment Among African American Breast Cancer Patients: One-Year Follow-Up Results of a Randomized Psychoeducational Group Intervention	RCT	73	Breast Cancer stage 0—IIIa	Not reported	Psychosocial support group	N/A	Control group	POMS, IES and MHI	Sig reduction in distress using POMS only (*p* < 0.24) Effect size: authors gave “small” (no figures) Data not provided to calculate confidence intervals
**Screening or assessment only**											
Braeken, APB [60]	2013	Psychosocial screening effects on health-related outcomes in patients receiving radiotherapy. A cluster RCT	Pragmatic Cluster RCT (Solomon four-group design)	568	Prostate/bladder, lung, breast, cervix/endometrium, rectum, non-Hodgkin’s	N 83.7% Unknown 16.3%	Psychological screening instrument (Dutch screening inventory (SIPP))	N/A	Usual care	HADS	Not sig
Carlson, LE [61]	2012	Online screening for distress, the 6th vital sign, in newly diagnosed oncology outpatients: RCT of computerised vs personalised triage	RCT	3133	All new cancers	Not reported	Computer Triage	N/A	Personalised Triage	DT	Sig reduction in distress *p* < 0.0001. Computer triage group had lower % participants with distress above the clinical cut-off (*p* < 0.05) at 3 months. Effect size: (gender and surgery subgroups: *d* < 0.15 (negligible)
Carlson, LE [62]	2010	Screening for Distress in Lung and Breast Cancer Outpatients: A Randomized Controlled Trial	RCT	1134	New breast or lung cancer	Y	Full Screening	Triage (full screening + included optional telephone triage)	Minimal screening (DT + usual care)	DT	Triage group had sig lower distress at 3 months (*p* = .031), controlling for baseline distress. Effect size: Full screen vs min screen d = 0.39 (small)^a^ CI: -0.88, 0.10^b^ Triage vs min screen d = -0.56^a^ CI: -1.06, -0.06^b^
Oerlemans, S [63]	2021	Web-Based Return of Individual Patient-Reported Outcome Results Among Patients with Lymphoma: Randomized Controlled Trial	RCT	227	Any lymphoma	Y	Return of patient reported outcome (PRO) results	PRO + living with lymphoma	Care as Usual (CAU)	HADS	Not sig
O'Hea, E [64]	2020	Impact of the mental health and dynamic referral for oncology (MHADRO) program on oncology patient outcomes, health care utilization, and health provider behaviors: A multi-site randomized control trial	RCT	836	Any cancer	Not reported	MHADRO + DR + provider report	N/A	MHADRO only	DT	Not sig
**Expressive or creative writing**											
de Moor, C [65]	2002	A Pilot Study of the Effects of Expressive Writing on Psychological and Behavioral Adjustment in Patients Enrolled in a Phase II Trial of Vaccine Therapy for Metastatic Renal Cell Carcinoma	Other: Random assignment using minimization	42	Metastatic Renal Cell Carcinoma	Y 76.2% had 2 or more metastases	Expressive Writing (IG)	N/A	Neutral Writing (CG)	IES	Not sig
Mosher, CE [66]	2012	Randomised trial of expressive writing for distressed metastatic breast cancer patients	RCT	90 baseline interviews. 87 randomised	Stage IV breast cancer	Y	Expressive writing	N/A	Neutral writing	DT	Not sig
Nesterova, D [67]	2022	Group-led creative writing and behavioural health in cancer: a randomised clinical trial	RCT	60	Any stage cancer	Y—Stage IV 38%	Creative Writing Workshop (CWW)	N/A	Active control (AC)	Emotion thermometer Scale (ETS)	Sig reduction in distress *p* < 0.0001. Withdrawal rate was high, reducing study power. Effect sizes not calc – no SDs provided. Distress in CWW group *p *= 0.0346 CI: -4.31, -2.47 (large)
Stanton, AL [68]	2002	Randomized, Controlled Trial of Written Emotional Expression and Benefit Finding in Breast Cancer Patients	RCT	63	Stage I or II breast cancer	N	EMO	POS	CTL	POMS and own ‘distress index’	Not sig
**Psychological and psychosocial therapies**											
Clark, PG [69]	2010	Decreasing Psychological Distress in Cancer Inpatients Using FLEX Care®: A Pilot Study	Other: Pre-post control group study	35	Any cancer diagnosis	Not clear 17.1% Stage IV 60% Stage not known	FLEX psychological intervention	N/A	Routine psychological intervention	BSI-18	Sig at *p* = 0.001 but small sample size and attrition of 26% resulted in an underpowered study and therefore effect sizes were not calculated
Mahendran, R [70]	2015	Efficacy of a brief nurse-led pilot psychosocial intervention for newly diagnosed Asian cancer patients	Quasi-experimental pilot study with patient self-selection of study group	121	Newly diagnosed first cancer	N	Brief nurse-led psychosocial intervention programme	N/A	Treatment as usual	DT	Sig reduction in distress *p* = 0.001 but at baseline intervention group had sig higher distress than control and therefore effect sizes are not meaningful
Semple, CJ [71]	2009	Development and evaluation of a problem-focused psychosocial intervention for patients with head and neck cancer	Quasi-experimental design patients self-selecting study group	54	Head and Neck Cancer	Y	Psychosocial (IG)	N/A	Usual Care (CG)	HADS	Sig in distress using HADS anxiety and depression scales (*p* = 0.001 and *p* = 0.005 respectively) Effect size: Anxiety d = -0.21 (small)^a^ CI: -3.53, 3.11^b^ Depression d = -1.5 (large)^a^ CI: -4.04, 1.04^b^
Wang, S [72]	2020	A Psychological Nursing Intervention for Patients with Thyroid Cancer on Psychological Distress and Quality of Life	RCT	268	Thyroid cancer	Y	Psychological nursing intervention (IG)	N/A	Routine care (CG)	POMS and HADS	Sig decrease in POMS TMD at 4 weeks *p* = 0.03 but not at 8 weeks (*p* = 0.08) Effect sizes: 8 weeks d = -0.2485 (small)^a^ CI: -0.4811, -0.0158^b^
**Dignity Therapy**											
Chochinov, HM [73]	2011	Effect of dignity therapy on distress and end-of-life experience in terminally ill patients: a RCT	RCT	441	Terminal cancer life expectancy â‰¤6 months	Y	Dignity Therapy (DT)	Client-Centred Care (CT)	Standard Care (SC)	Palliative Performance Scale, PDI, HADS	Not sig
Hall, S [74]	2011	A novel approach to enhancing hope in patients with advanced cancer: a randomised phase II trial of dignity therapy	RCT	45	Any advanced cancer	Not reported	Dignity therapy	N/A	Control group (usual care)	PDI	Not sig
Li, Y-C [75]	2020	The Effectiveness of Dignity Therapy as Applied to End-of-Life Patients with Cancer in Taiwan: A Quasi-Experimental Study	Other: quasi experimental non RCT	30	Any end-stage cancer	Y	Dignity Therapy	N/A	Control group	PDI	Not sig
Vuksanovic, D [76]	2017	Dignity Therapy and Life Review for Palliative Care Patients: A Randomized Controlled Trial	RCT	70	Terminal disease	Not reported	Dignity Therapy (DT)	Life Review (LR)	Waitlist Control (WC)	PDI	Not sig
**Web-based or mobile app interventions**											
Çınar, D [77]	2021	Effect of mobile phone app-based training on the quality of life for women with breast cancer	Other: Randomised pre-post-test design	64	Non-metastatic breast cancer	N	Mobile app-based education (IG)	N/A	Control group—assessment only (CG)	DT	Sig reduction in distress at 12 weeks (*p* < 0,05). ANOVA significant (F = 11,214, *p* = 0,001) Effect size: d = -0.56 (mod) CI: -1.0635, -0.0638
de Hosson, LD [78]	2019	Web-based personalised information and support for patients with a neuroendocrine tumour: RCT	RCT	105	NET	Not reported	Web-based, personalised information and support system (WINS)	N/A	Standard Care	DT	Not sig
Salzer, MS [79]	2010	A randomized, controlled study of Internet peer-to-peer interactions among women newly diagnosed with breast cancer	RCT	78	Breast cancer stage I-II	N	Internet peer support (IG)	N/A	Internet-based control condition (CG)	HSCL-25 and IES	Not sig
**Life Review**											
Chen, Y [80]	2020	Effects of a mind map-based life review programme on psychospiritual well-being in cancer patients undergoing chemotherapy: A RCT	RCT	84	Cancer (any)	Y 88.09% N 11.91%	Mind map-based life review programme (MBLRP) + routine care	N/A	Routine care	DT	Not sig
Sun, FK [81]	2021	The Effects of Logotherapy on Distress, Depression, and Demoralization in Breast Cancer and Gynecological Cancer Patients	Other: Quasi experimental (pre-test, post-test)	64	Breast, ovarian, cervical or endometrial cancer	Unclear 22% stage 3 or above	Logotherapy	N/A	Control—education session	DT	Not sig
Xiao, H [82]	2013	Effect of a Life Review Program for Chinese Patients with Advanced Cancer	RCT	80	Advanced cancer of any type	Y	Life Review Programme	N/A	Control group	QoL existential distress subscale	Not sig
**Problem-solving approaches, education and information**											
Nezu, AM [83]	2003	Project Genesis: Assessing the Efficacy of Problem-Solving Therapy for Distressed Adult Cancer Patients	RCT	150	Cancer	N	Problem-solving therapy (PST)	PST for patient and significant other PST-SO)	Waiting-list control (WLC)	POMS	Sig reduction in distress at 6 months and 1-year post-intervention *p* < 0.001 Effect size: d = 2.17 (large) patients only d = 2.04 (large) for patients + significant other (*p* < 0.001). Rate of improvement = 67% and 59% respectively
Passalacqua, R [84]	2009	Prospective, Multicenter, Randomized Trial of a New Organizational Modality for Providing Information and Support to Cancer Patients	Other: pragmatic, two-arm cluster randomized trial	38 oncology centres and 3,197 patients	Any cancer	Not reported	Point of Information and Support (PIS)	N/A	No PIS	HADS	Not sig
Sandgren, AK [85]	2007	Long-term telephone therapy outcomes for breast cancer patients	RCT	218	Breast cancer: stages I-III	N	Health education therapy (IG1)	Emotional expression therapy (IG2)	Control group (CG)	POMS	Sig reduction in distress at 0–6 months and 6–13 months in ALL groups including control. Therefore, no significant intervention effect
**Couples’ (dyadic) therapies**											
Manne, SL [86]	2019	Couple-focused interventions for men with localized prostate cancer and their spouses: A randomized clinical trial	RCT	237	Localised prostate cancer	N	Intimacy enhancing therapy (IG1)	General health and wellness intervention (IG2)	Usual care CG	IES	Not sig
Manne, SL [87]	2016	A Randomized Clinical Trial of a Supportive versus a Skill-Based Couple-Focused Group Intervention for Breast Cancer Patients	Other: Randomised clinical trial, no usual control group	302 couples (604 people)	Breast cancer	N (up to stage 3a)	Couples-focused support group (ECG)	Support Group (SG)	No control group	IES	Sig reductions in distress for SG group for most distressed pts. Sig reductions in distress in ECG group for less distressed pts (*p* < 0.01). Effect sizes: given as between d = 0.29 and 0.55 (small – moderate) [CIs not given]
**Physical Therapies**											
Araújo, RV [88]	2021	Effect of Raja Yoga Meditation on the Distress and Anxiety Levels of Women with Breast Cancer	RCT	50	Breast cancer	Y	Raja Yoga meditation	N/A	Educational activity	DT	Sig reduction in distress post-intervention *p* < 0.001 Effect size: d = 1.49 (large) [CIs not given]
Kovacic, T [89]	2011	Impact of Relaxation Training According to Yoga in Daily Life® System on Perceived Stress After Breast Cancer Surgery	RCT	32	Breast cancer stage I or II	N (Stage III and IV excluded)	Physiotherapy plus Yoga in Daily Life (YIDL)	N/A	Standard physiotherapy	RSCL (psych subscale) and GHQ-12	Sig reduction in distress at 4 weeks using RSCL *p* < 0.0005. GHQ-12 sig at *p* < 0.05. Effect sizes: GHQ-12: d = -17.57^a^ CI: -20.13, -15.01 (large)^b^ RSCL = -16.50^a^ CI: -18.37, -14.63 (large)^b^
**Art or music therapies**											
Hanser, SB [90]	2006	Effects of a Music Therapy Intervention on Quality of Life and Distress in Women with Metastatic Breast Cancer	RCT	70	Metastatic breast cancer	Y	Music Therapy (MT)	N/A	Usual care	HADS	Not sig
Radl, D [91]	2018	The effects of Self-Book© art therapy on cancer-related distress in female cancer patients during active treatment: A randomized controlled trial	RCT	60	Any cancer site	Stage IV Self-Book—30% Standard care—20%	Self-book	N/A	Standard care	DT	Not sig
**Others (uncategorised)**											
Eychmüller S [92]	2021	Single early palliative care intervention added to usual oncology care for patients with advanced cancer: A randomized controlled trial (SENS Trial)	RCT	150	Lung, colorectal, prostate, breast, urothelial, pancreatic	Metastatic 99% Usual care 97%	Early palliative care intervention (IG)	N/A	Usual care (CG)	DT	Not sig
Ferrell, B [93]	2021	A Palliative Care Intervention for Patients on Phase 1 Studies	RCT	479	Solid tumour cancer	Not reported	Palliative care intervention	N/A	Usual care	DT	Sig reduction in distress at site 1 (-2.03 points on scale *p* < 0.001) but not at site 2 (-0.26 points, *p* = 0.80) but site 1 nurses were more experienced
Grégoire, C [94]	2018	Efficacy of a hypnosis-based intervention to improve well-being during cancer: a comparison between prostate and breast cancer patients	CCT	92 breast and 42 prostate—total = 138	Non-metastatic breast or prostate cancer	N	Self-hypnosis/self-care (SH)	N/A	Usual care	HADS anxiety and depression	Prostate: not sig Breast: Sig reduction in distress *p* = 0.031. Group sig reductions *p* = 0.023 (but women had higher scores at baseline) Effect sizes: d = 0.66 anxiety (mod) d = 0.47 depression (low)
Grégoire, C [95]	2017	Group interventions to reduce emotional distress and fatigue in breast cancer patient: a 9-month follow-up pragmatic trial	CCT; Pragmatic design	138	Non-metastatic breast cancer	N	Yoga	Self -hypnosis/self care	CBT and 4th group Control (usual care)	HADS	Not sig for CBT. Sig for self-hypnosis (anxiety and depression) (both *p* = 0.000) and yoga (anxiety only) (*p* = 0.024) with sig time- effects as well Effect sizes: NOT GIVEN
Han, X-B [96]	2021	Efficacy of combined naikan and morita therapies on psychological distress and posttraumatic growth in Chinese patients with advanced cancer. A randomized controlled trial	RCT	130	Stage III or IV breast, lung, colorectal or renal cancer	Stage IV Treatment 30.77% Control 27.69%	Naikan/Morita program	N/A	Usual Care control (CG)	DT	Sig at *p* < 0.001 at immediate post-treatment Effect sizes: d = -2.39 (large)^a^ CI: -2.86, -1.92
Schuurhuizen CSEW [97]	2019	Screening and Stepped Care Targeting Psychological Distress in Patients with Metastatic Colorectal Cancer: The TES Cluster Randomized Trial	Other: Cluster RCT	349	Metastatic colorectal cancer	Y	Screening and Stepped Care (TES) programme	N/A	Care as Usual (CAU)	HADS	Not sig
Young, JM [98]	2013	Multicenter Randomized Trial of Centralized Nurse-Led Telephone-Based Care Coordination to Improve Outcomes After Surgical Resection for Colorectal Cancer: The CONNECT Intervention	RCT	775	Colorectal cancer	Y	CONNECT telephone intervention	N/A	Usual care	FACT-C and DT	Not sig
Young, J [99]	2010	Development and feasibility assessment of telephone-delivered supportive care to improve outcomes for patients with colorectal cancer: pilot study of the CONNECT intervention	Other: Prospective non-randomised trial	41	Colorectal cancer	Y	CONNECT intervention	N/A	Control group	FACT-C and DT	Not sig

Key:

*CBT* Cognitive behavioural therapy, *DT* Distress thermometer, *HADS* Hospital Anxiety and Depression Scale, *BSI-18* Brief Symptom Inventory-18, *IES* Impact of Events Scale, *SCL-90r* Symptom Checklist-90 revised, *ETS* Emotional Thermometer Scale, *FACT-C* Functional Assessment of Cancer Therapy-Colorectal, *GHQ-12* General Health Questionnaire, *HSCL-25* Hopkins Symptom Checklist, *K10* Kessler physiological distress scale, *PDI* Patient dignity inventory, *POMS* Profile of Mood States, *QoL* Quality of life, *MHI – 17-item* Mental Health Inventory, *RSCL* Rotterdam Symptom Checklist, *PSS-10* Perceived stress scale, *TAU* treatment-as-usual, *CI* Confidence Interval, *d* = Cohen’s measure of effect size

^a^Effect size calculated by the authors for purposes of the systematic review [100]

^b^Confidence interval (CI) calculated by the authors for purposes of the systematic review [100]

Of the 59 included studies, 45 (78%) were randomised controlled trials (RCTs) two were CCTs and 12 were classed as ‘others’ and included cluster designs, pragmatic trials and quasi-experimental controlled studies. None of the trials were described as mixed-methodology studies although some did contain minimal qualitative data. Twenty-four studies (41%) were based in the USA or Europe. Participant characteristics between studies were variable by gender, type and stage of cancer, including patients in the early stages of cancer through to those in palliative care. Across all included studies the total number of participants randomised (in RCTs) or consented (in CCTs) was 17,628. The number of participants per study ranged from 30 to 3133 (including cluster studies and dyads) and the mean number of participants per study was 298.1 (median 122).

A high degree of heterogeneity was evident across the included studies in relation to the interventions, dose, the outcome measures used and follow-up times. Seventeen different measures of distress were used in the included studies. Not all these measures have been specifically validated for cancer populations.

Criteria suggested by Borenstein, et al., was used to decide whether pooling data for a meta-analysis was appropriate [101]. These criteria include a subjective assessment of the similarity of studies in terms of patients, inclusion criteria and baseline characteristics, and comparing studies with the same interventions, comparators and outcomes. Only three RCTs met the criteria for similarity of patients, inclusion criteria, baseline characteristics and outcome measures [43, 44, 47] and all were higher quality studies as evaluated by MMAT [37]. However, Liu, et al. [44] had less than 80% adherence to the intervention and Park, et al. [47] aborted recruitment before the target was reached. There was also uncertainty/doubt as to the method of randomisation used in Compen, et al. [43]. These 3 studies also looked at different cancer types and stages. Patients were recruited in different ways with notable differences in gender proportions. Also, the total number of pooled events from these 3 studies would have been < 500 which, according to Moore, et al. [39, 102] would be insufficient. Heterogeneity and small numbers of studies therefore precluded meta-analysis.

### Quality analysis and risk of bias

The MMAT tool for quality assessment was used independently by two authors (EC and CP). Any disparities were discussed and agreed (Appendix D(b) (Supplementary information)). Of the 59 included studies, 35 (59.3%) lacked outcome assessor blinding and 27 (45.8%) studies had < 80% adherence to the intervention. An important finding was that 33 (55.9%) studies did not report any sample-size calculation so there was no indication of statistical power. Also, 53 (89.8%) of studies did not record the presence or absence of adverse events. Failure to record and report adverse events is an important omission, especially in advanced cancer, because some interventions may result in greater distress due to an increased focus and attention of the patient on their disease and its associated problems (Paley CA: Investigations into the use of acupuncture for treating cancer-induced bone pain in adults, unpublished).

Other study design and quality issues included a lack of explanation regarding randomisation methods and some did not report whether study arms had comparable demographics and baseline measurements.

In terms of methodological quality, only three studies, Araújo, et al., [88] Compen, et al. [43] and Semple, et al. [71] met all the basic MMAT criteria but did not positively meet the additional questions added regarding reporting adverse events, and although Semple et al. did calculate sample size, this study was not an RCT and patients self-selected their study arm, thus introducing bias. Only the study by Araújo, et al. [88] positively met all the MMAT criteria and additional questions but was still a relatively small study with only 50 participants in total. Semple et al. [71] was also a small study with 54 participants. Compen, et al. [43] had a larger sample of 245 participants, but these were randomised to 3 arms: face-to-face- group MBCT, internet-based eMBCT and treatment as usual (TAU). Overall, the methodological quality of studies included in this review was low, mainly due to small sample-sizes and a lack of outcome assessor blinding in more than one third of studies. Unclear reporting and baseline differences in study groups were also prevalent.

#### The evidence for reductions in cancer-related distress

For ease of reference, the included studies were divided into broad intervention groups: mindfulness, talking/communication/CBT/group therapies, screening/assessment only, expressive/creative writing, psychological/psychosocial therapies, dignity therapy, web-based/mobile app, life review, problem-solving/education, couples (dyadic) therapies, physical therapies, art/music and others (uncategorised) (Table 1).

Of the 59 included studies, 29 (54.2%) reported statistically significant reductions in psychological distress at follow-up. The remaining 30 studies did not find that the interventions made any significant changes in distress. Within the studies reporting significant changes were three anomalies: the study by Sandgren, et al. [85] used telephone therapy, but both intervention groups and control group showed a similar decline in levels of distress; Mahendran, et al. [70] compared a brief psychosocial intervention with a control condition, but the level of distress in the intervention group was significantly higher at baseline so the results were skewed in favour of the intervention; and Clark, et al., [69] had a small sample size (*n* = 35) with a 26% attrition rate, leaving a small and under-powered study.

Of the remaining 26 studies showing statistically significant intervention effects, not all included effect sizes or provided data from which these could be calculated. Where data was available, effect sizes were calculated using Cohen’s *d,* (standardised mean difference), however, it is important to acknowledge that effect sizes are only meaningful for comparison if there is certainty that compared studies are similar in study design [103]. Cohen’s *d* is conventionally regarded as small at 0.2 or less, 0.5 as medium, and 0.8 as large [104], although these definitions are somewhat arbitrary [105, 106].

The mindfulness category was the largest group comprising variations on one specific approach (mindfulness) and included nine studies. All nine were RCTs and six out of nine studies showed positive effects, reaching statistical significance. Only one of these studies by Park, et al., had a large effect size (*d* = -1.03; [CI: -1.51 to -0.54] (*p* < *0.001*)) at 12 weeks post-intervention when mindfulness-based cognitive therapy (MBCT) was compared with a waiting list control group in breast cancer patients [47], although recruitment was aborted before the target sample size was reached and there was no assessor blinding. Four studies had moderate effect-sizes [43, 44, 46, 48] and of these, Compen, et al. had the largest sample size and compared face to face mindfulness training (MBCT) and online MBCT (eMBCT) against a treatment-as-usual (TAU) control group. This was a relatively high-quality study, meeting all MMAT criteria and with a small effect size of *d* = *-*0.45 [95% CI: -0.83, -0.14 (*p* < *0.001*)] for MBCT but reaching a moderate effect size *d* = *-*0.71 [95% CI: -1.04, -0.37 (*p* < *0.001*)] for eMBCT. All mindfulness studies with a moderate or large effect-size used the Hospital Anxiety and Depression Scale (HADS) as a primary outcome measure, although variation in methodologies was still present, which precluded meta-analysis. One other study in the mindfulness category with statistically significant improvements in distress had a small effect size (*d* = -0.43) and did not provide data with which to calculate confidence intervals [49].

There were ten studies within the talking/communication/group therapies category. In this broad group, six studies out of ten reported significant intervention-related reductions in distress [50–59]. Mertz, et al. had a large effect size for screening and counselling [58], however this was a pilot study and had a small sample size (*n* = 50) with only 41 participants completing the intervention. Quality assessment using MMAT showed that the randomisation method for this study was unclear and there was no evidence of assessor blinding or of a sample-size calculation. Hejazi, et al. also demonstrated a large effect size for a communication programme in elderly cancer patients but again, the uncalculated sample size was small (*n* = 64), randomisation methodology was unclear and there was no outcome assessor blinding. They also omitted to report adverse events. A study by Chambers, et al. had a moderate effect size for web-based cognitive behavioural therapy (CBT) [55] but did not have comparable groups in the study arms, failed to report outcome assessor blinding and ≤ 80% of participants adhered to the intervention. Two other studies in this group showed no significant effects on distress. A pragmatic trial by Gregoire, et al. [95], which was categorised in the ‘others’ group, because it had multiple arms of different interventions, should be mentioned here because it had a CBT arm. This found no statistically significant changes in distress following the intervention, although it was significant for yoga and self-hypnosis. The CBT study arm also had very few participants (*n* = 10).

Within this category there were there were three statistically significant group interventions for distress [51, 52, 59]. The two studies by Andersen, et al. [51, 52] did not provide a full data set. The study by Taylor, et al. [59] described the effect size as “small”, with no figures provided. This study also had a small sample size and did not meet many of the MMAT quality standards, with no blinding, incomplete outcome data and fewer than 80% of participants completing the intervention.

Of all the other categories, there were two other studies with large effect sizes. Nezu, et al. [83] used problem-solving therapy with (PST) and without (PST-SO) a significant other accompanying the patient, compared with a waiting-list control. Both interventions were significant for reduction in distress immediately post-intervention, at 6 months and 1 year using POMS as an outcome measure. The effect sizes calculated for the purposes of this systematic review at post-intervention were large: *d* = 1.993 (PST) and *d* = 1.643 (PST-SO), and at 12 months: *d* = 2.17 and *d* = 2.04 respectively (all *P* < 0.001). Nevertheless, the study was of low quality as the authors did not report outcome assessor blinding or sample size calculation and the study had a small number of participants. Kovačič, et al. [89] used a yoga intervention compared with standard physiotherapy only but although the effect sizes were large the study was very small with only 32 patients in total. No sample-size calculation was carried out.

Other studies meeting MMAT criteria, coupled with significant improvements in distress, included a yoga intervention (Araújo, et al. [88]) and a psychosocial intervention (Semple, et al. [71]) but the sample sizes for both studies were very small. Han, et al. [96] compared Naikan and Morita therapy [107] against usual care (*n* = 130) and had a large effect size immediately post-treatment for improvements in distress amongst Chinese breast cancer patients. Naikan and Morita therapy has Japanese/Buddhist roots and requires absolute commitment from the patient who puts him/herself under the total direction of the therapist.

The remaining studies across categories used a variety of interventions in various combinations and in different settings, including face-to-face therapies and web-or app-based interventions. Patient groups varied from all cancers at all stages to specific cancer sites or specified stages; e.g., stages I-III or stage IV metastatic cancer.

## Discussion

### Summary of main findings

This systematic review has shown a wide variation in approaches to alleviating distress in patients with cancer. There was no definitive consensus on any one intervention or means of delivery, although therapies involving mindfulness-based approaches were the most frequently researched with some evidence of efficacy, followed by talking or communication-based therapies and interventions conducted in groups with weaker evidence of benefit. In this review, mindfulness interventions were generally of high methodological quality. However, as stated in a systematic review and meta-analysis by Faller, et al., many studies with large effect sizes mostly have small sample sizes which will tend to inflate effect size estimation and should therefore be interpreted with caution [28].

### Implications of this review

Only three studies from the 26 showing statistically significant intervention-related reductions in distress were rated positive on all MMAT quality standards, and of these, the largest study (Compen, et al. [43]) used MBCT compared with eMBCT and TAU, was the most promising. However, both intervention groups were resource-heavy, requiring input from trained therapists; particularly the face-to-face group which included 8 weekly group sessions along with home practice. The other two high quality studies with significant benefits for distress involved a yoga intervention (Araújo, et al. [88]) and a psychosocial intervention (Semple, et al. [71]); again, both resource-heavy in terms of staff training and time. In a recent unpublished survey of hospices in England, it was identified that healthcare professionals perceive there to be substantial gaps in training and supervision for meeting the psychological assessment and treatment needs of patients [108]. The survey identified wide variations and major gaps in provision of psychological screening/assessment and intervention across this sector, which suggests that resource-heavy interventions do not represent a practical way forward.

Taking resources into consideration, brief interventions for distress, especially those which can be self-administered after training, are likely to be the most feasible in practice. In 2020, Compen, et al., looked at the cost-effectiveness of mindfulness-based cognitive therapy, either online or face to face, compared with treatment as usual [109]. They revealed positive findings, especially for an internet-based intervention, which also has the advantage of convenience for patients and staff. Mindfulness interventions can be very brief (as little as 5 min) and can be taught relatively quickly to patients and their families or carers so that the techniques can be used flexibly, as required. Such interventions which allow self-management of symptoms have potential to be widely incorporated into routine care. Mindfulness techniques have also been found to improve motivation for the adoption of healthy lifestyle changes and enhancement of interpersonal relationships [110]. Evidence suggests that patients prefer self-help strategies to manage their distress when needed [111], but there is little evidence supporting self-guided interventions and further evidence is needed to either support the efficacy of current strategies or suggest new ones [112].

Of the studies showing benefit, nine interventions were under 1 h duration and sessions continued for 1 month or less, but only three of these demonstrated statistically significant improvements in distress [46, 56, 62]. One of these studies used an intervention that was described as ‘brief’ (5 min of mindfulness) [46]. In the studies showing no significant effect on psychological distress there was another 5 min mindful breathing intervention which demonstrated significant and rapid reductions in ‘perceived stress’, rather than distress [42]. Six other interventions were delivered on an as-needed basis or self-managed. These findings were similar to another systematic review by Xunlin, et al., [31] which included 29 randomised controlled trials (RCTs) of mindfulness interventions to improve quality of life in patients with cancer. Their review showed that mindfulness techniques are effective in reducing anxiety, depression, and stress in cancer patients and survivors.

### Comparison with other systematic reviews

Other meta-analyses have specifically examined the effects of mindfulness-based stress reduction techniques, and these show significant effects on distress [28, 33, 113]. Cillessen, et al. included 29 RCTs with a total of 3274 patients [33]. It demonstrated small, but significant treatment effects for follow-up of up to 6 months when a manual for the intervention was followed and when patient groups were younger (mean age 55 years), compared with a passive control group [33]. A meta-analysis conducted by Haller, et al., also found significant effects of mindfulness-based interventions on health-related quality of life, fatigue, sleep, stress, anxiety, and depression in women with breast cancer although the effect sizes were small [32]. Faller, et al., conducted a systematic review and meta-analysis of psycho-oncologic interventions for emotional distress and quality of life in adults with cancer and concluded that there were small to medium effect sizes for individual, group, and couples psychotherapy, psychoeducation, and relaxation training, but there were methodological shortcomings including study quality and risk of bias [28]. Most studies incorporated CBT techniques, usually in combination with other techniques, such as coping strategies, but none used mindfulness to alleviate distress.

It has been suggested that because CBT techniques are commonly used for distress in cancer patients and have found to be effective [114], it might be useful to conduct research using both techniques in combination [33]. Our review did not reveal many studies using CBT as a single technique, although it was used in combination with mindfulness in three studies, as MBCT and Park, et al. reported large effect sizes for the intervention and Compen et al. demonstrated moderate effects [41, 43, 47]. As discussed above, implementation of CBT is more resource-dependent, usually requiring face-to-face contact.

As previously described, Warth, et al., conducted a similar review but only included patients in the advanced stages of terminal illness (not necessarily cancer) with a prognosis of < 3 months [30], which precluded direct comparisons. Also, the interventions included were only those with ≤ 4 sessions and < 21 days. Four of the papers met the inclusion criteria for our review [46, 74, 76, 82]. Warth, et al. demonstrated significant effects on emotional and existential distress and quality of life. However, the authors acknowledged a number of limitations, including baseline differences, a generally low methodological quality and possibly an underpowered meta-regression analysis. The authors did not examine follow-up data. They concluded that that psychosocial techniques are effective, and that these include interventions such as mindfulness, dignity therapy, life review, and creative-based therapies.

It was interesting that none of our included studies investigated acceptance and commitment therapy (ACT), as it has recently become more widely used and this was highlighted in a cross-sectional survey of therapeutic approaches used in UK hospices [115]. A systematic review investigating the use of ACT for psychological and physical symptoms amongst cancer patients revealed large effect sizes on psychological distress in cancer patients, although this was predominantly in younger patients who lived in eastern countries and received therapy for longer [27].

An important consideration is the country of origin for each study. Those carried out in regions where healthcare has to be paid for (e.g., the USA or parts of the far east) might be biased in favour of patients who had access to cancer treatment and were therefore recruited into research studies during clinic appointments. In addition, cultural differences make comparisons of interventions problematic.

High attrition rates are frequently a problem in cancer-related studies, particularly where patients are in the advanced stages of the disease with a high symptom burden or where patients lack social support from family and friends [116, 117]. Some studies have recorded drop-out rates of up to 50% [116]. This might suggest that brief interventions for distress, and particularly those which can be self-administered as needed, would be more practical and have better adherence, especially in patients who are in the advanced stages of cancer.

### Limitations of this review

This review had a number of limitations. The inclusion criteria restricted the review to studies published in the English language and our searches only included published literature. The inclusion criteria were very broad across study methodologies and populations to enable identification of as many relevant studies as possible. This added complexity when comparing the efficacy of studies. The resulting heterogeneity of studies precluded meta-analysis. Restricting our searches to RCTs would have enabled us to use the Cochrane Risk of Bias tool (ROB2) and the GRADE quality assessment rather than the MMAT tool which did not provide as much sensitivity for RCTs. However, this would have narrowed the scope of the review and not given such a wide picture of the range of interventions being used for psychological distress and how they were delivered. Also, excluding studies involving cancer survivors, according to our narrower definition of survivors as those not undergoing active treatment may have resulted in some relevant interventions, such as ACT, being missed; although each title, abstract and full text was examined by two authors. Nevertheless, the review has revealed some interesting and useful information which has allowed us to suggest some implications for clinical practice and possible directions for future research.

### Implications for clinical practice

Mindfulness interventions appear to be effective and appropriate for people with cancer, particularly those with advanced disease. Mindfulness techniques are relatively quick to teach, and can be self-administered outside medical settings by patients and carers. They can be taught face to face, via the internet and practised at home by patients or carers who have had some instruction. We suggest that brief mindfulness interventions, might also be suitable for use when needed in palliative and end-of-life care when patients are often unable to cope with more lengthy interventions or activities requiring sustained concentration.

### Implications for research

Further and more robust evidence is required to support the findings of this review. A clear international consensus of psychological distress needs to be established, along with core, validated outcome measures. Studies should be adequately powered and of high methodological quality to reduce bias and provide reliable evidence-based guidance for those working with this patient group. There is a growing body of evidence to indicate that mindfulness interventions are beneficial to patients, and feasible to implement and utilise. Future studies should focus on the efficacy of self-administered, brief mindfulness interventions for psychological distress in patients with advanced disease.

## Conclusions

The majority of studies using mindfulness interventions in this review are efficacious at alleviating distress. We suggest that brief mindfulness interventions might be appropriate for clinical implementation in advanced disease and palliative care. Our review suggests that therapist-guided or online interventions show greater efficacy in reducing distress but self-directed mindfulness interventions have merit in by allowing patients to use these techniques when needed. In conclusion, mindfulness interventions merit further investigation using adequately powered, high-quality studies.

## Supplementary Information

**Additional file 1: Appendix A.** Search strategies. **Appendix B.** PRISMA Checklist. **Appendix C.** Covidence Data Extraction Template QR Code. **Appendix D(a).** MMAT Criteria. **Appendix D(b).** MMAT Scores.

## Data Availability

Review data are available from corresponding author; C. A. Paley (c.a.paley@leeds.ac.uk).

## Abbreviations

ACT: Acceptance and commitment therapy
AMA: American Medical Association
CBT: Cognitive behavioural therapy
CCT: Controlled Clinical Trial
DT: Distress thermometer
eMBCT: Online (electronic) mindfulness-based cognitive therapy
GRADE: Grading of Recommendations, Assessment, Development, and Evaluations
HADS: Hospital Anxiety and Depression Scale
MBCT: Mindfulness-based cognitive therapy
MBSR: Mindfulness-based stress reduction
MMAT: Mixed methods appraisal tool
NCCN: National Comprehensive Cancer Network
PST: Problem-solving therapy with a significant other
PST-SO: Problem-solving therapy without a significant other
POMS: Profile of mood states
PRISMA: Preferred Reporting Items for Systematic Reviews and Meta-Analyses
RCT: Randomised controlled trial
RoB2: Cochrane risk of bias tool
TAU: Treatment as usual
UC: Usual care

## References

[1] Huda N, Shaw MK, Chang HJ (2022). Psychological Distress Among Patients With Advanced Cancer: A Conceptual Analysis. Cancer Nurs.

[2] NCCN Clinical Practice Guidelines in Oncology (NCCN Guidelines®) for Distress Management V.2.2023. © National Comprehensive Cancer Network, Inc. 2023. NCCN.org. Accessed 10 May 2023.

[3] LeBlanc TW, Kamal AH (2017). Assessing psychological toxicity and patient-reported distress as the sixth vital sign in cancer care and clinical trials. AMA J Ethics.

[4] IPOS. IPOS international standard of quality cancer care, https://www.ipos-society.org/about/quality (2010). Accessed 19 July 2022.

[5] Watson M, Bultz BD (2010). Distress, the 6th vital sign in cancer care. Psycho-Oncologie.

[6] Ridner SH (2004). Psychological distress: concept analysis. J Adv Nurs.

[7] Walker L, Avant K. Concept analysis. Strategies for theory construction in nursing. 6th ed. NY: Pearson; 2019.

[8] Chapman EJ, Pini S, Edwards Z, et al. Conceptualising effective symptom management in palliative care: a novel model derived from qualitative data. BMC Palliative Care. 2022; 21. 10.1186/s12904-022-00904-9. 10.1186/s12904-022-00904-9 PMC8815221 35115005

[9] Gao W, Bennett MI, Stark D (2010). Psychological distress in cancer from survivorship to end of life care: prevalence, associated factors and clinical implications. Eur J Cancer.

[10] Deshields TL, Wells-Di Gregorio S, Flowers SR (2021). Addressing distress management challenges: recommendations from the consensus panel of the american psychosocial oncology society and the association of Oncology social work. CA Cancer J Clin.

[11] Kaye J, Gracely E (1993). Psychological distress in cancer patients and their spouses. J Cancer Educ.

[12] Gröpper S, van der Meer E, Landes T (2016). Assessing cancer-related distress in cancer patients and caregivers receiving outpatient psycho-oncological counseling. Support Care Cancer.

[13] Segrin C, Badger TA (2010). Psychological distress in different social network members of breast and prostate cancer survivors. Res Nurs Health.

[14] Bultz BD, Carlson LE (2006). Emotional distress: the sixth vital sign–future directions in cancer care. Psychooncology.

[15] Mehnert A, Hartung TJ, Friedrich M (2018). One in two cancer patients is significantly distressed: prevalence and indicators of distress. Psycho-oncology (Chichester, England).

[16] Clover KA, Mitchell AJ, Britton B (2015). Why do oncology outpatients who report emotional distress decline help?. Psychooncology.

[17] Carolan CM, Smith A, Davies GR (2018). Seeking, accepting and declining help for emotional distress in cancer: A systematic review and thematic synthesis of qualitative evidence. Eur J Cancer Care.

[18] Corrigan P (2004). How stigma interferes with mental health care. Am Psychol.

[19] Holland JC, Alici Y (2010). Management of distress in cancer patients. J Support Oncol.

[20] McCarter K, Britton B, Baker AL (2018). Interventions to improve screening and appropriate referral of patients with cancer for psychosocial distress: systematic review. BMJ Open.

[21] Holland J (1999). NCCN practice guidelines for the management of psychosocial distress. Natl Comprehen Cancer Network Oncol.

[22] Ziegler L, Hill K, Neilly L (2011). Identifying psychological distress at key stages of the cancer illness trajectory: a systematic review of validated self-report measures. J Pain Symptom Manage.

[23] Butow P, Shaw J, Shepherd HL (2018). Comparison of implementation strategies to influence adherence to the clinical pathway for screening, assessment and management of anxiety and depression in adult cancer patients (ADAPT CP): study protocol of a cluster randomised controlled trial. BMC Cancer.

[24] Butow P, Shepherd HL, Cuddy J (2021). Acceptability and appropriateness of a clinical pathway for managing anxiety and depression in cancer patients: a mixed methods study of staff perspectives. BMC Health Serv Res.

[25] Watson L, Groff S, Tamagawa R (2016). Evaluating the impact of provincial implementation of screening for distress on quality of life, symptom reports, and psychosocial well-being in patients with cancer. J Natl Compr Canc Netw.

[26] Troy JD, de Castro CM, Pupa MR (2018). Patient-reported distress in myelodysplastic syndromes and its association with clinical outcomes: a retrospective cohort study. J Natl Compr Canc Netw.

[27] Zhao C, Lai L, Zhang L (2021). The effects of acceptance and commitment therapy on the psychological and physical outcomes among cancer patients: A meta-analysis with trial sequential analysis. J Psychosom Res.

[28] Faller H, Schuler M, Richard M (2013). Effects of psycho-oncologic interventions on emotional distress and quality of life in adult patients with cancer: systematic review and meta-analysis. J Clin Oncol.

[29] Lepore SJ, Coyne JC (2006). Psychological interventions for distress in cancer patients: a review of reviews. Ann Behav Med.

[30] Warth M, Kessler J, Koehler F (2019). Brief psychosocial interventions improve quality of life of patients receiving palliative care: A systematic review and meta-analysis. Palliat Med.

[31] Xunlin NG, Lau Y, Klainin-Yobas P (2020). The effectiveness of mindfulness-based interventions among cancer patients and survivors: a systematic review and meta-analysis. Support Care Cancer.

[32] Haller H, Winkler MM, Klose P (2017). Mindfulness-based interventions for women with breast cancer: an updated systematic review and meta-analysis. Acta Oncol.

[33] Cillessen L, Johannsen M, Speckens AEM (2019). Mindfulness-based interventions for psychological and physical health outcomes in cancer patients and survivors: a systematic review and meta-analysis of randomized controlled trials. Psychooncology.

[34] Page MJ, McKenzie JE, Bossuyt PM (2021). The PRISMA 2020 statement: an updated guideline for reporting systematic reviews. BMJ.

[35] Page MJ, Moher D, Bossuyt PM (2021). PRISMA 2020 explanation and elaboration: updated guidance and exemplars for reporting systematic reviews. BMJ.

[36] Veritas. Covidence systematic review software. 2022. www.covidence.org. Accessed 24 Mar 2022.

[37] Hong QN, Gonzalez-Reyes A, Pluye P (2018). Improving the usefulness of a tool for appraising the quality of qualitative, quantitative and mixed methods studies, the Mixed Methods Appraisal Tool (MMAT). J Eval Clin Pract.

[38] Higgins JPT, Thomas J, Chandler J, Cumpston M, Li T, Page MJ, Welch VA, editors. Cochrane Handbook for Systematic Reviews of Interventions version 6.3 (updated February 2022). Cochrane; 2022. Available from www.training.cochrane.org/handbook.

[39] Moore AR, Gavaghan D, Tramèr RM (1998). Size is everything–large amounts of information are needed to overcome random effects in estimating direction and magnitude of treatment effects. Pain.

[40] Moore RA, Derry S, McQuay HJ (2010). Clinical effectiveness: an approach to clinical trial design more relevant to clinical practice, acknowledging the importance of individual differences. Pain.

[41] Chambers SK, Occhipinti S, Foley E (2017). Mindfulness-Based Cognitive Therapy in Advanced Prostate Cancer: A Randomized Controlled Trial. J Clin Oncol.

[42] Chui PL, Wai S, Lai LL (2021). Mindful Breathing: Effects of a Five-Minute Practice on Perceived Stress and Mindfulness Among Patients With Cancer. Clin J Oncol Nurs.

[43] Compen F, Bisseling E, Schellekens M (2018). Face-to-face and internet-based mindfulness-based cognitive therapy compared with treatment as usual in reducing psychological distress in patients with cancer: a multicenter randomized controlled trial. J Clin Oncol.

[44] Liu Z, Li M, Jia Y (2022). A randomized clinical trial of guided self-help intervention based on mindfulness for patients with hepatocellular carcinoma: effects and mechanisms. Jpn J Clin Oncol.

[45] Milbury K, Li Y, Durrani S (2020). A mindfulness-based intervention as a supportive care strategy for patients with metastatic non-small cell lung cancer and their spouses: results of a three-arm pilot randomized controlled trial. Oncologist.

[46] Ng CG, Lai KT, Tan SB (2016). The effect of 5 minutes of mindful breathing to the perception of distress and physiological responses in palliative care cancer patients: a randomized controlled study. J Palliat Med.

[47] Park S, Sato Y, Takita Y (2020). Mindfulness-based cognitive therapy for psychological distress, fear of cancer recurrence, fatigue, spiritual well-being, and quality of life in patients with breast cancer-a randomized controlled trial. J Pain Symptom Manage.

[48] Schellekens MPJ, van den Hurk DGM, Prins JB (2017). Mindfulness-based stress reduction added to care as usual for lung cancer patients and/or their partners: A multicentre randomized controlled trial. Psychooncology.

[49] Wurtzen H, Dalton SO, Christensen J (2015). Effect of mindfulness-based stress reduction on somatic symptoms, distress, mindfulness and spiritual wellbeing in women with breast cancer: Results of a randomized controlled trial. Acta Oncol.

[50] Acevedo-Ibarra JN, Juárez-García DM, Espinoza-Velazco A (2019). Cognitive Behavioral Stress Management intervention in Mexican colorectal cancer patients: Pilot study. Psycho-Oncology.

[51] Andersen BL, Farrar WB, Golden-Kreutz D (2007). Distress reduction from a psychological intervention contributes to improved health for cancer patients. Brain Behav Immun.

[52] Andersen BL, Farrar WB, Golden-Kreutz DM (2004). Psychological, behavioral, and immune changes after a psychological intervention: a clinical trial. J Clin Oncol.

[53] Boesen EH, Karlsen R, Christensen J (2011). Psychosocial group intervention for patients with primary breast cancer: a randomised trial. Eur J Cancer.

[54] Boesen EH, Ross L, Frederiksen K (2005). Psychoeducational intervention for patients with cutaneous malignant melanoma: a replication study. J Clin Oncol.

[55] Chambers SK, Ritterband LM, Thorndike F (2018). Web-delivered cognitive behavioral therapy for distressed cancer patients: randomized controlled trial. J Med Internet Res.

[56] Hejazi F, Bahrami M, Keshvari M (2017). The effect of a communicational program on psychological distress in the elderly suffering from cancer. Iran J Nurs Midwifery Res.

[57] Manne SL, Virtue SM, Ozga M (2017). A comparison of two psychological interventions for newly-diagnosed gynecological cancer patients. Gynecol Oncol.

[58] Mertz BG, Dunn-Henriksen AK, Kroman N (2017). The effects of individually tailored nurse navigation for patients with newly diagnosed breast cancer: a randomized pilot study. Acta Oncol.

[59] Taylor KL, Lamdan RM, Siegel JE (2003). Psychological adjustment among African American breast cancer patients: One-year follow-up results of a randomized psychoeducational group intervention. Health Psychol.

[60] Braeken AP, Kempen GI, Eekers DB (2013). Psychosocial screening effects on health-related outcomes in patients receiving radiotherapy A cluster randomised controlled trial. Psychooncology.

[61] Carlson LE, Waller A, Groff SL (2012). Online screening for distress, the 6th vital sign, in newly diagnosed oncology outpatients: randomised controlled trial of computerised vs personalised triage. Br J Cancer.

[62] Carlson LE, Groff SL, Maciejewski O (2010). Screening for distress in lung and breast cancer outpatients: a randomized controlled trial. J Clin Oncol.

[63] Oerlemans S, Arts LPJ, Kieffer JM (2021). Web-based return of individual patient-reported outcome results among patients with lymphoma: randomized controlled trial. J Med Internet Res.

[64] O'Hea E, Kroll-Desrosiers A, Cutillo AS (2020). Impact of the mental health and dynamic referral for oncology (MHADRO) program on oncology patient outcomes, health care utilization, and health provider behaviors: A multi-site randomized control trial. Patient Educ Counsel.

[65] de Moor C, Sterner J, Hall M (2002). A pilot study of the effects of expressive writing on psychological and behavioral adjustment in patients enrolled in a Phase II trial of vaccine therapy for metastatic renal cell carcinoma. Health Psychol.

[66] Mosher CE, DuHamel KN, Lam J (2012). Randomised trial of expressive writing for distressed metastatic breast cancer patients. Psychol Health.

[67] Nesterova D, Zhu J, Kramer C, et al. Group-led creative writing and behavioural health in cancer: a randomised clinical trial. BMJ Support Palliat Care. 2021. 10.1136/bmjspcare-2020-002463 10.1136/bmjspcare-2020-002463 33423021

[68] Stanton AL, Danoff-Burg S, Sworowski LA (2002). Randomized, controlled trial of written emotional expression and benefit finding in breast cancer patients. J Clin Oncol.

[69] Clark PG (2010). Decreasing psychological distress in cancer inpatients using FLEX Care®: a pilot study. Soc Work Health Care.

[70] Mahendran R, Lim HA, Tan JYS (2015). Efficacy of a brief nurse-led pilot psychosocial intervention for newly diagnosed Asian cancer patients. Support Care Cancer.

[71] Semple CJ, Dunwoody L, Kernohan WG (2009). Development and evaluation of a problem-focused psychosocial intervention for patients with head and neck cancer. Support Care Cancer.

[72] Wang S, Huang H, Wang L (2020). A psychological nursing intervention for patients with thyroid cancer on psychological distress and quality of life: a randomized clinical trial. J Nerv Ment Dis.

[73] Chochinov HM, Kristjanson LJ, Breitbart W (2011). Effect of dignity therapy on distress and end-of-life experience in terminally ill patients: a randomised controlled trial. Lancet Oncol.

[74] Hall S, Goddard C, Opio D (2011). A novel approach to enhancing hope in patients with advanced cancer: a randomised phase II trial of dignity therapy. BMJ Support Palliat Care.

[75] Li YC, Feng YH, Chiang HY (2020). The effectiveness of dignity therapy as applied to end-of-life patients with cancer in Taiwan: a quasi-experimental study. Asian Nurs Res (Korean Soc Nurs Sci).

[76] Vuksanovic D, Green HJ, Dyck M (2017). Dignity Therapy and Life Review for Palliative Care Patients: A Randomized Controlled Trial. J Pain Symptom Manage.

[77] Cinar D, Karadakovan A, Erdogan AP (2021). Effect of mobile phone app-based training on the quality of life for women with breast cancer. Eur J Oncol Nurs.

[78] De Hosson LD, Bouma G, Stelwagen J (2019). Web-based personalised information and support for patients with a neuroendocrine tumour: Randomised controlled trial. Orphanet J Rare Dis.

[79] Salzer MS, Palmer SC, Kaplan K (2010). A randomized, controlled study of Internet peer-to-peer interactions among women newly diagnosed with breast cancer. Psychooncology.

[80] Chen Y, Xiao H, Zheng J (2020). Effects of a mind map-based life review programme on psychospiritual well-being in cancer patients undergoing chemotherapy: a randomised controlled trial. Eur J Cancer Care (Engl).

[81] Sun F-K, Hung C-M, Yao Y (2021). The effects of logotherapy on distress, depression, and demoralization in breast cancer and gynecological cancer patients: a preliminary study. Cancer Nurs.

[82] Xiao H, Kwong E, Pang S (2013). Effect of a life review program for Chinese patients with advanced cancer: a randomized controlled trial. Cancer Nurs.

[83] Nezu AM, Nezu CM, Felgoise SH (2003). Project Genesis: assessing the efficacy of problem-solving therapy for distressed adult cancer patients. J Consult Clin Psychol.

[84] Passalacqua R, Caminiti C, Campione F (2009). Prospective, multicenter, randomized trial of a new organizational modality for providing information and support to cancer patients. J Clin Oncol.

[85] Sandgren AK, McCaul KD (2007). Long-term telephone therapy outcomes for breast cancer patients. Psychooncology.

[86] Manne SL, Kashy DA, Zaider T (2019). Couple-focused interventions for men with localized prostate cancer and their spouses: A randomized clinical trial. Br J Health Psychol.

[87] Manne SL, Siegel SD, Heckman CJ (2016). A randomized clinical trial of a supportive versus a skill-based couple-focused group intervention for breast cancer patients. J Consult Clin Psychol.

[88] Araujo RV, Fernandes AFC, Campelo RCV (2021). Effect of raja yoga meditation on the distress and anxiety levels of women with breast cancer. Religions.

[89] Kovačič T, Kovačič M (2011). Impact of relaxation training according to Yoga In Daily Life® system on perceived stress after breast cancer surgery. Integr Cancer Ther.

[90] Hanser SB, Bauer-Wu S, Kubicek L (2006). Effects of a music therapy intervention on quality of life and distress in women with metastatic breast cancer. J Soc Integr Oncol.

[91] Radl D, Vita M, Gerber N (2018). The effects of Self-Book© art therapy on cancer-related distress in female cancer patients during active treatment: A randomized controlled trial. Psychooncology.

[92] Eychmuller S, Zwahlen S, Fliedner MC (2021). Single early palliative care intervention added to usual oncology care for patients with advanced cancer: A randomized controlled trial (SENS Trial). Palliat Med.

[93] Ferrell B, Chung V, Hughes MT (2021). A palliative care intervention for patients on phase 1 studies. J Palliat Med.

[94] Gregoire C, Nicolas H, Bragard I (2018). Efficacy of a hypnosis-based intervention to improve well-being during cancer: a comparison between prostate and breast cancer patients. BMC Cancer.

[95] Gregoire C, Bragard I, Jerusalem G (2017). Group interventions to reduce emotional distress and fatigue in breast cancer patients: a 9-month follow-up pragmatic trial. Br J Cancer.

[96] Han XB, Fang YQ, Liu SX (2021). Efficacy of combined naikan and morita therapies on psychological distress and posttraumatic growth in Chinese patients with advanced cancer: A randomized controlled trial. Medicine (Baltimore).

[97] Schuurhuizen C, Braamse AMJ, Beekman ATF (2019). Screening and stepped care targeting psychological distress in patients with metastatic colorectal cancer: the TES cluster randomized trial. J Natl Compr Cancer Netw.

[98] Young JM, Butow PN, Walsh J (2013). Multicenter randomized trial of centralized nurse-led telephone-based care coordination to improve outcomes after surgical resection for colorectal cancer: the CONNECT intervention. J Clin Oncol.

[99] Young J, Harrison J, Solomon M (2010). Development and feasibility assessment of telephone-delivered supportive care to improve outcomes for patients with colorectal cancer: pilot study of the CONNECT intervention. Support Care Cancer.

[100] Review Manager (RevMan) [Computer program]. Version 5.4. The Cochrane Collaboration; 2020.

[101] Borenstein L, Hedges L, Higgins J, et al. Ch. 45. When does it make sense to perform a meta-analysis? In: Introduction to meta-analysis. 2nd ed: Wiley; 2021. p. 393–400.

[102] Moore AR, Eccleston C, Derry S (2010). “Evidence” in chronic pain–establishing best practice in the reporting of systematic reviews. Pain.

[103] McGough JJ, Faraone SV (2009). Estimating the size of treatment effects: moving beyond p values. Psychiatry (Edgmont).

[104] Cohen J. Statistical power analysis for the behavioral sciences. Burlington: Elsevier Science; 2013. https://search.ebscohost.com/login.aspx?direct=true&scope=site&db=nlebk&db=nlabk&AN=923159. Accessed July 10 2022

[105] Lakens D (2013). Calculating and reporting effect sizes to facilitate cumulative science: a practical primer for t-tests and ANOVAs. Front Psychol.

[106] Thompson B (2007). Effect sizes, confidence intervals, and confidence intervals for effect sizes. Psychol Sch.

[107] Murase T, Johnson F (1974). Naikan, Morita, and Western psychotherapy: A comparison. Arch Gen Psychiatry.

[108] Paley CA, Arango MF, Keshwala V (2022). Evaluating provision of psychological assessment and support in palliative care: A survey of hospices in England.

[109] Compen F, Adang E, Bisseling E (2020). Cost-utility of individual internet-based and face-to-face Mindfulness-Based Cognitive Therapy compared with treatment as usual in reducing psychological distress in cancer patients. Psychooncology.

[110] Ludwig DS, Kabat-Zinn J (2008). Mindfulness in medicine. Jama.

[111] Clover KA, Mitchell AJ, Britton B, et al. Why do oncology outpatients who report emotional distress decline help? Psycho-Oncology. 2015;24:812–8. 20141211. 10.1002/pon.3729. 10.1002/pon.3729 25504987

[112] Ugalde A, Haynes K, Boltong A (2016). Self-guided interventions for managing psychological distress in people with cancer—A systematic review. Patient Educ Couns.

[113] Zhang Q, Zhao H, Zheng Y (2019). Effectiveness of mindfulness-based stress reduction (MBSR) on symptom variables and health-related quality of life in breast cancer patients-a systematic review and meta-analysis. Support Care Cancer.

[114] Tatrow K, Montgomery GH (2006). Cognitive behavioral therapy techniques for distress and pain in breast cancer patients: a meta-analysis. J Behav Med.

[115] McInnerney D, Candy B, Stone P (2021). Access to and adequacy of psychological services for adult patients in UK hospices: a national, cross-sectional survey. BMC Palliat Care.

[116] Gebert P, Schindel D, Frick J (2021). Characteristics and patient-reported outcomes associated with dropout in severely affected oncological patients: an exploratory study. BMC Med Res Methodol.

[117] Bennett MI, Johnson MI, Brown SR (2010). Feasibility study of Transcutaneous Electrical Nerve Stimulation (TENS) for cancer bone pain. J Pain.

