# Supplementary material for: Non-pharmacological interventions to manage psychological distress in patients living with cancer: a systematic review
Source: BMC Palliat Care. 2023 Jul 6;22:88. doi: 10.1186/s12904-023-01202-8 (PMC10324164; doi:10.1186/s12904-023-01202-8)
Supplement: Supplementary file 1 — Additional file 1: Appendix A. Search strategies. Appendix B. PRISMA Checklist. Appendix C. Covidence Data Extraction Template QR Code. Appendix D(a). MMAT Criteria. Appendix D(b). MMAT Scores. [file 12904_2023_1202_MOESM1_ESM.docx]

## **Appendix A: Search strategies**

| **Research question:** | What interventions are used to address psychological distress in patients with cancer and which show efficacy? | | | | |
| --- | --- | --- | --- | --- | --- |
| **Places to search for information:** | 1. MEDLINE (via OVID) 2. APA PsycINFO (OVID) 3. AMED (OVID) 4. Web of Science 5. CINAHL (via EBSCO) | | 1. Scopus 2. PubMed   CENTRAL (Cochrane)  Other systematic reviews  Original scoping review | | |
| **List of sources searched:** | **Date of search** | **Search strategy used, including any limits** | | **Total references** | **Comments** |
| OVID: Medline, APA PsychInfo and AMED | 06/04/2022 | Distress$.mp. [mp=ab, hw, ti, bt, ot, nm, fx, kf, ox, px, rx, an, ui, sy, tc, id, tm, mf] Cancer.mp. [mp=ab, hw, ti, bt, ot, nm, fx, kf, ox, px, rx, an, ui, sy, tc, id, tm, mf] Psychological intervention.mp. [mp=ab, hw, ti, bt, ot, nm, fx, kf, ox, px, rx, an, ui, sy, tc, id, tm, mf] Limit 3 to all journals Limit 4 to english language Limit 5 to human Remove duplicates | | **164** | Searches were de-duplicated by OVID |
| Web of Science | 06/04/2022 | (((((((TS=(Distress)) AND TS=(cancer)) AND TS=(psychological distress)) AND TS=(clinical trial)) NOT TS=(cancer survivor)) NOT TS=(children)) NOT TS=(paediatric)) NOT TS=(pediatric)  Exclusions: book articles, conference proceedings/abstracts, letters, editorial materials. | | **304** |  |
| CINAHL (EBSCO) | 06/04/2022 | ( Distress AND ( cancer patients or oncology patients or patients with cancer or neoplasms ) AND ( psychological distress ) AND ( clinical study or clinical trial ) ) NOT ( children or adolescents or youth or child or teenager )  Limiters – English Language; Published Date: 2002-2022; Human(s); Adults. | | **282** |  |
| Scopus | 06/04/2022 | Distress AND cancer AND NOT survivors AND NOT children  AND  ( LIMIT-TO ( PUBYEAR ,  2002-2022 )  AND  LIMIT-TO ( DOCTYPE ,  "ar" )  AND  LIMIT-TO ( EXACTKEYWORD ,  "Human" )  OR  LIMIT-TO ( EXACTKEYWORD ,  "Humans" )  OR  LIMIT-TO ( EXACTKEYWORD ,  "Adult" ) )  AND  LIMIT-TO ( LANGUAGE ,  "English" ) | | **63** | Also applied limits to inappropriate subject areas |
| PUBMED | 06/04/2022 | ((((Distress[Title/Abstract]) AND (cancer[Title/Abstract])) AND (psychological distress[Title/Abstract])) NOT (Children[Title/Abstract])) NOT (Survivor[Title/Abstract]) Filters: Clinical Trial, Randomized Controlled Trial, from 2002/1/1 - 2022/4/6 | | **317** | Also applied limits to inappropriate subject areas |
| Cochrane CENTRAL database | 31/05/2022 | *Cancer in Keyword AND Distress in Keyword AND "controlled clinical trial" in Keyword AND Intervention in Keyword NOT Children in Keyword | | **11** |  |
| Refs identified from original scoping review checked for additional papers | 26/05/2022 | (DISTRESS[Title/Abstract]) AND (CANCER[Title/Abstract]) AND (INTERVENTION[Title/Abstract])) NOT (CHILDREN[Title/Abstract]) NOT (survivors)  Total = 1237 refs identified | | **31** |  |
| Additional references from other SRs | 30/05/2022 | All refs in SRs added if meeting inclusion criteria | | **15** |  |
|  |  | **TOTAL References imported to Covidence** | | **1187** |  |

## **Appendix B: PRISMA Checklist**

| **Section and Topic** | **Item #** | **Checklist item** | **Location where item is reported** |
| --- | --- | --- | --- |
| **TITLE** | | |  |
| Title | 1 | Identify the report as a systematic review. | Title page |
| **ABSTRACT** | | |  |
| Abstract | 2 | See the PRISMA 2020 for Abstracts checklist. | Abstract |
| **INTRODUCTION** | | |  |
| Rationale | 3 | Describe the rationale for the review in the context of existing knowledge. | Introduction |
| Objectives | 4 | Provide an explicit statement of the objective(s) or question(s) the review addresses. | Introduction pg 6/7 |
| **METHODS** | | |  |
| Eligibility criteria | 5 | Specify the inclusion and exclusion criteria for the review and how studies were grouped for the syntheses. | Pg 7/8 |
| Information sources | 6 | Specify all databases, registers, websites, organisations, reference lists and other sources searched or consulted to identify studies. Specify the date when each source was last searched or consulted. | Pg 8 |
| Search strategy | 7 | Present the full search strategies for all databases, registers and websites, including any filters and limits used. | Pg 8/9 & Appendix |
| Selection process | 8 | Specify the methods used to decide whether a study met the inclusion criteria of the review, including how many reviewers screened each record and each report retrieved, whether they worked independently, and if applicable, details of automation tools used in the process. | Pg 9/10 |
| Data collection process | 9 | Specify the methods used to collect data from reports, including how many reviewers collected data from each report, whether they worked independently, any processes for obtaining or confirming data from study investigators, and if applicable, details of automation tools used in the process. | Pg 9/10 |
| Data items | 10a | List and define all outcomes for which data were sought. Specify whether all results that were compatible with each outcome domain in each study were sought (e.g. for all measures, time points, analyses), and if not, the methods used to decide which results to collect. | Pg 7/8 |
|  | 10b | List and define all other variables for which data were sought (e.g. participant and intervention characteristics, funding sources). Describe any assumptions made about any missing or unclear information. | Pg 7/8 |
| Study risk of bias assessment | 11 | Specify the methods used to assess risk of bias in the included studies, including details of the tool(s) used, how many reviewers assessed each study and whether they worked independently, and if applicable, details of automation tools used in the process. | Pg 10 |
| Effect measures | 12 | Specify for each outcome the effect measure(s) (e.g. risk ratio, mean difference) used in the synthesis or presentation of results. | Pg 10 |
| Synthesis methods | 13a | Describe the processes used to decide which studies were eligible for each synthesis (e.g. tabulating the study intervention characteristics and comparing against the planned groups for each synthesis (item #5)). | Pg 12/13 & pg 25/26 |
|  | 13b | Describe any methods required to prepare the data for presentation or synthesis, such as handling of missing summary statistics, or data conversions. | Pg 9/10 & Pg 25 |
|  | 13c | Describe any methods used to tabulate or visually display results of individual studies and syntheses. | Pg 9/10 & Pg 25 |
|  | 13d | Describe any methods used to synthesize results and provide a rationale for the choice(s). If meta-analysis was performed, describe the model(s), method(s) to identify the presence and extent of statistical heterogeneity, and software package(s) used. | Pg 12/13 & pg 25/26 |
|  | 13e | Describe any methods used to explore possible causes of heterogeneity among study results (e.g. subgroup analysis, meta-regression). | N/A |
|  | 13f | Describe any sensitivity analyses conducted to assess robustness of the synthesized results. | N/A |
| Reporting bias assessment | 14 | Describe any methods used to assess risk of bias due to missing results in a synthesis (arising from reporting biases). | Pg 10 & pg 13 |
| Certainty assessment | 15 | Describe any methods used to assess certainty (or confidence) in the body of evidence for an outcome. | P9 9/10 & Pg 25/26 |
| **RESULTS** | | |  |
| Study selection | 16a | Describe the results of the search and selection process, from the number of records identified in the search to the number of studies included in the review, ideally using a flow diagram. | Pg 10-13 |
|  | 16b | Cite studies that might appear to meet the inclusion criteria, but which were excluded, and explain why they were excluded. | N/A |
| Study characteristics | 17 | Cite each included study and present its characteristics. | Table 1, pg 14-24 |
| Risk of bias in studies | 18 | Present assessments of risk of bias for each included study. | Appendices |
| Results of individual studies | 19 | For all outcomes, present, for each study: (a) summary statistics for each group (where appropriate) and (b) an effect estimate and its precision (e.g. confidence/credible interval), ideally using structured tables or plots. | Pg 14-24 |
| Results of syntheses | 20a | For each synthesis, briefly summarise the characteristics and risk of bias among contributing studies. | Pg 25-29 |
|  | 20b | Present results of all statistical syntheses conducted. If meta-analysis was done, present for each the summary estimate and its precision (e.g. confidence/credible interval) and measures of statistical heterogeneity. If comparing groups, describe the direction of the effect. | Pg 25-29 |
|  | 20c | Present results of all investigations of possible causes of heterogeneity among study results. | Pg 12,13,26 & 32 |
|  | 20d | Present results of all sensitivity analyses conducted to assess the robustness of the synthesized results. | N/A |
| Reporting biases | 21 | Present assessments of risk of bias due to missing results (arising from reporting biases) for each synthesis assessed. | Pg 25-29 |
| Certainty of evidence | 22 | Present assessments of certainty (or confidence) in the body of evidence for each outcome assessed. | Table 1, pg 14-24 pg 26 |
| **DISCUSSION** | | |  |
| Discussion | 23a | Provide a general interpretation of the results in the context of other evidence. | Pg 29-34 |
|  | 23b | Discuss any limitations of the evidence included in the review. | Pg 32/33 |
|  | 23c | Discuss any limitations of the review processes used. | Pg 32/33 |
|  | 23d | Discuss implications of the results for practice, policy, and future research. | Pg 33/34 |
| **OTHER INFORMATION** | | |  |
| Registration and protocol | 24a | Provide registration information for the review, including register name and registration number, or state that the review was not registered. | Title page |
|  | 24b | Indicate where the review protocol can be accessed, or state that a protocol was not prepared. | Title page |
|  | 24c | Describe and explain any amendments to information provided at registration or in the protocol. | N/A |
| Support | 25 | Describe sources of financial or non-financial support for the review, and the role of the funders or sponsors in the review. | Pg 36 |
| Competing interests | 26 | Declare any competing interests of review authors. | Pg 36/37 |
| Availability of data, code and other materials | 27 | Report which of the following are publicly available and where they can be found: template data collection forms; data extracted from included studies; data used for all analyses; analytic code; any other materials used in the review. | Pg 9 |

*From:*  Page MJ, McKenzie JE, Bossuyt PM, Boutron I, Hoffmann TC, Mulrow CD, et al. The PRISMA 2020 statement: an updated guideline for reporting systematic reviews. BMJ 2021;372:n71. doi: 10.1136/bmj.n71

For more information, visit: <http://www.prisma-statement.org/>

## **Appendix C: Covidence Data Extraction Template QR Code**


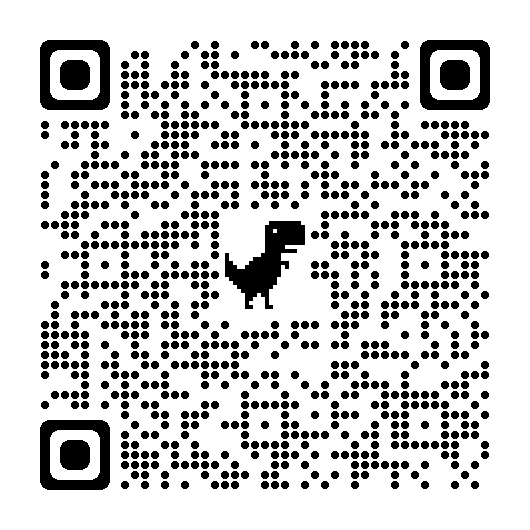


## **Appendix D(a): MMAT Criteria**


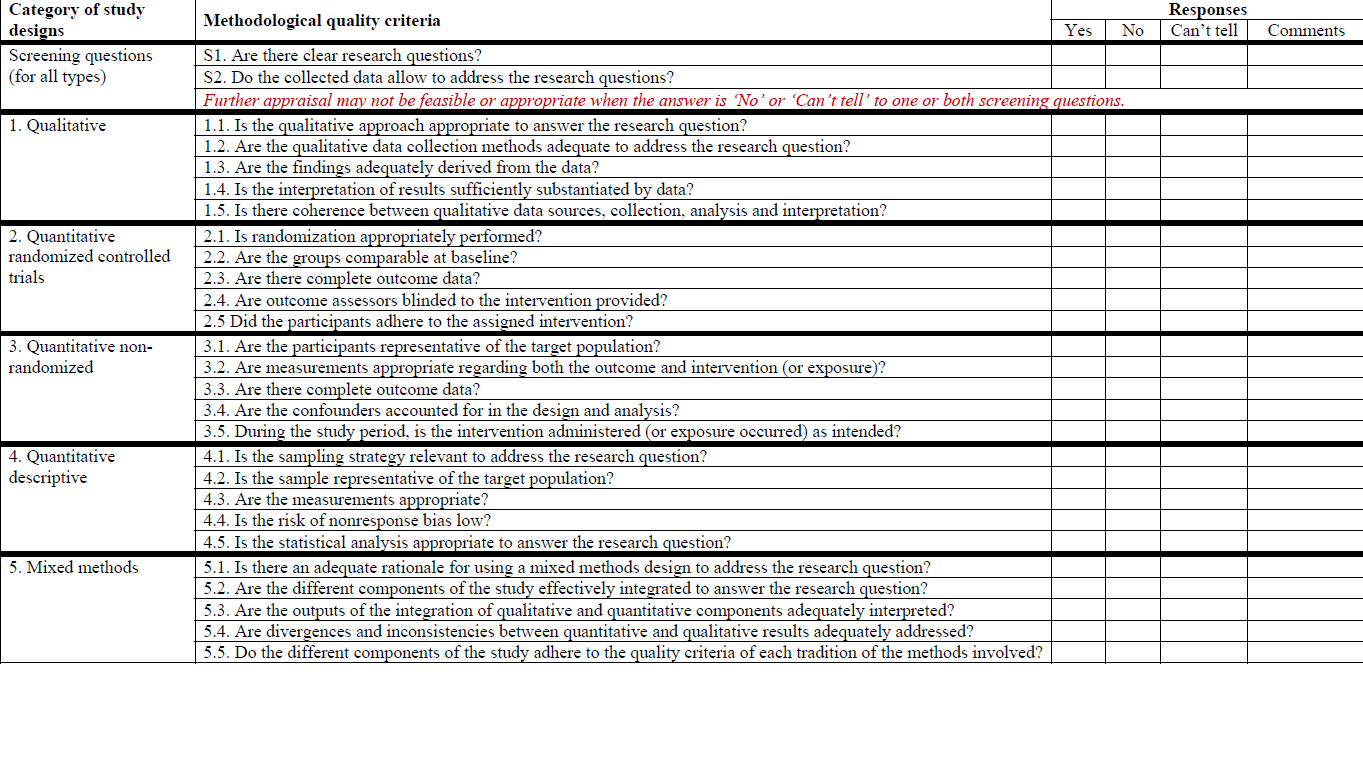


## **Appendix D(b): MMAT Scores**

| **Study identifiers** | | **Screening** | | | **RCT quantitative design only** | | | | | | **Non-Randomised designs only** | | | | | | **Decisions** |
| --- | --- | --- | --- | --- | --- | --- | --- | --- | --- | --- | --- | --- | --- | --- | --- | --- | --- |
| **Author** | **Publication date** | **Screening 1** | **Screening 2** | **Study Design** | | **Randomisation appropriately performed?** | **Comparable groups?** | **Complete outcome data?** | **Outcome assessor blinding?** | **Did participants adhere to the intervention? ≥80%** | | **Participants representative of target population?** | **Are measures appropriate for both the outcome and intervention or exposure?** | **Complete outcome data?** | **Are confounders accounted for in the design and analysis?** | **Is the intervention administered (or exposure occurred) as intended?** | **MMAT comments on quality appraisal** |
| Acevedo-Ibarra | 2019 | Y | Y | RCT | | Y | Y | Y | Y | N | |  |  |  |  |  | Pilot study. |
| Andersen | 2007 | Y | Y | RCT | | Y | Y | N | Y | Y | |  |  |  |  |  |  |
| Andersen | 2004 | Y | Y | RCT | | Y | Y | Y | U | Y | |  |  |  |  |  |  |
| Araújo | 2021 | Y | Y | RCT | | Y | Y | Y | Y | Y | |  |  |  |  |  | **Yes for all basic MMAT criteria** |
| Boesen | 2011 | Y | Y | RCT | | Y | Y | Y | Y | Y | |  |  |  |  |  | **Yes for all basic MMAT criteria** |
| Boesen | 2005 | Y | Y | RCT | | Y | Y | Y | N | Y | |  |  |  |  |  |  |
| Braeken | 2013 | Y | Y | RCT (cluster) | | Y | Y | Y | N | Y | |  |  |  |  |  |  |
| Carlson | 2010 | Y | Y | RCT | | Y | Y | Y | Y (Partial) | U | |  |  |  |  |  | Blinding was partial |
| Carlson | 2012 | Y | Y | RCT | | Y | Y | Y | Y (Partial) | N | |  |  |  |  |  |  |
| Chambers | 2017 | Y | Y | RCT | | Y | Y | Y | N | N | |  |  |  |  |  | 58% adherence at last follow-up |
| Chambers | 2018 | Y | Y | RCT | | Y | N | Y | N | N | |  |  |  |  |  | Post-hoc sample size calculation. |
| Chen | 2020 | Y | Y | RCT | | Y | Y | Y | Y | Y | |  |  |  |  |  |  |
| Chochinov | 2011 | Y | Y | RCT | | Y | Y | N (see comments) | Y | N | |  |  |  |  |  | No baseline distress measures |
| Chui | 2021 | Y | Y | RCT | | Y | Y | Y | N | Y | |  |  |  |  |  |  |
| Çınar | 2021 | Y | Y | RCT | | U | Y | Y | N | U | |  |  |  |  |  |  |
| Clark | 2010 | Y | Y | RCT | | U | Y | Y | N | N | |  |  |  |  |  | Pilot study |
| Compen | 2018 | Y | Y | RCT | | Y | Y | Y | Y | Y | |  |  |  |  |  | **Yes for all basic MMAT criteria** |
| de Hosson | 2019 | Y | Y | RCT | | Y | U | Y | N | Y | |  |  |  |  |  | Pilot study |
| de Moor | 2002 | Y | Y | RCT | | Y | Y | Y | N | Y | |  |  |  |  |  |  |
| Eychmüller | 2021 | Y | Y | RCT | | Y | Y | Y | Y | N | |  |  |  |  |  |  |
| Sun | 2021 | Y | Y | CCT | |  |  |  |  |  | | Y | Y | Y | Y | Y | **Yes for all basic MMAT criteria** |
| Ferrell | 2021 | Y | Y | RCT | | Y | Y | Y | N | N | |  |  |  |  |  |  |
| Grégoire | 2017 | Y | Y | CCT (Pragmatic) | |  |  |  |  |  | | Y | Y | Y | Y | Y | **Yes for all basic MMAT criteria** |
| Grégoire | 2018 | Y | Y | CCT | |  |  |  |  |  | | Y | Y | Y | Y | Y | **Yes for all basic MMAT criteria** |
| Hall | 2011 | Y | Y | RCT | | Y | U | Y | N | N | |  |  |  |  |  |  |
| Hanser | 2006 | Y | Y | RCT | | Y | Y | Y | N | N | |  |  |  |  |  |  |
| Han | 2021 | Y | Y | RCT | | Y | Y | Y | N | Y | |  |  |  |  |  |  |
| Hejazi | 2017 | Y | Y | RCT | | U | Y | Y | N | Y | |  |  |  |  |  |  |
| Kovacic | 2011 | Y | Y | RCT | | Y | U | Y | Y | U | |  |  |  |  |  |  |
| Liu | 2022 | Y | Y | RCT | | Y | Y | Y | N | N | |  |  |  |  |  |  |
| Li | 2020 | Y | Y | CCT | |  |  |  |  |  | | Y | Y | Y | Y | Y | **Yes to all basic MMAT criteria.** |
| Mahendran | 2015 | Y | Y | CCT | |  |  |  |  |  | | Y | Y | N | Y | N |  |
| Manne | 2019 | Y | Y | RCT | | U | U | Y | N | N | |  |  |  |  |  |  |
| Manne | 2016 | Y | Y | RCT | | Y | N | Y | N | N | |  |  |  |  |  |  |
| Manne | 2017 | Y | Y | RCT | | Y | Y | Y | N | N - only up to T5 then 75.6% | |  |  |  |  |  |  |
| Mertz | 2017 | Y | Y | RCT | | U | U | Y | N | Y | |  |  |  |  |  | Pilot study |
| Milbury | 2020 | Y | Y | RCT | | Y | U | N | N | N | |  |  |  |  |  |  |
| Mosher | 2012 | Y | Y | RCT | | Y | Y | Y | N | Y | |  |  |  |  |  |  |
| Nesterova | 2022 | Y | Y | RCT | | U | U | N - see comments | N | N | |  |  |  |  |  | Outcome data were not accessible. |
| Nezu | 2003 | Y | Y | RCT | | Y | Y | Y | N | N | |  |  |  |  |  |  |
| Ng | 2016 | Y | Y | RCT | | U | U | Y | N | Y | |  |  |  |  |  |  |
| Oerlemans | 2021 | Y | Y | RCT | | Y | Y | Y | N | N - only up to 4 month follow-up | |  |  |  |  |  |  |
| O'Hea | 2020 | Y | Y | RCT | | U | Y | Y | Y | N | |  |  |  |  |  |  |
| Park | 2020 | Y | Y | RCT | | Y | Y | Y | N | Y | |  |  |  |  |  | Recruitment aborted before target sample size reached |
| Passalacqua | 2009 | Y | Y | RCT | | U | Y | Y | N | Y | |  |  |  |  |  |  |
| Radl | 2018 | Y | Y | RCT | | Y | Y | Y | N | N | |  |  |  |  |  |  |
| Salzer | 2010 | Y | Y | RCT | | U | Y | Y | N | Y | |  |  |  |  |  | Pilot study |
| Sandgren | 2007 | Y | Y | RCT | | Y | U | Y | N | U | |  |  |  |  |  |  |
| Schellekens | 2017 | Y | Y | RCT | | Y | Y | Y | N | N | |  |  |  |  |  |  |
| Schuurhuizen | 2019 | Y | Y | RCT | | U | Y | Y | Y | N - 80% compliant until 10 wk | |  |  |  |  |  |  |
| Semple | 2009 | Y | Y | CCT | |  |  |  |  |  | | Y | Y | Y | Y | Y | **Yes to basic MMAT criteria** |
| Shujia | 2020 | Y | Y | RCT | | U | Y | Y | Y | Y | |  |  |  |  |  |  |
| Stanton | 2002 | Y | Y | RCT | | Y | U | Y | Y | Y | |  |  |  |  |  |  |
| Taylor | 2003 | Y | Y | RCT | | U | Y | N | N | N | |  |  |  |  |  |  |
| Vuksanovic | 2017 | Y | Y | RCT | | Y | Y | Y | N | Y | |  |  |  |  |  |  |
| Wurtzen | 2015 | Y | Y | RCT | | Y | Y | Y | N | Y | |  |  |  |  |  |  |
| Xiao | 2013 | Y | Y | RCT | | Y | Y | Y | N | N | |  |  |  |  |  |  |
| Young | 2010 | Y | Y | CCT | |  |  |  |  |  | | Y | Y | Y | Y | Y | **Yes to basic MMAT criteria** |
| Young | 2013 | Y | Y | RCT | | Y | U | Y | Y | Y | |  |  |  |  |  | **Yes to basic MMAT criteria** |
|  |  |  |  |  | |  |  |  | N=35 | N=27 | |  |  |  |  |  |  |
